# Late effects of total body irradiation on hematopoietic recovery and immune function in rhesus macaques

**DOI:** 10.1371/journal.pone.0210663

**Published:** 2019-02-13

**Authors:** Laura P. Hale, Gowrisankar Rajam, George M. Carlone, Chen Jiang, Kouros Owzar, Greg Dugan, David Caudell, Nelson Chao, J. Mark Cline, Thomas C. Register, Gregory D. Sempowski

**Affiliations:** 1 Department of Pathology and Duke Human Vaccine Institute, Duke University School of Medicine, Durham, NC, United States of America; 2 Immunobiology Laboratory, Centers for Disease Control and Prevention (CDC), Atlanta, GA, United States of America; 3 Duke Cancer Institute, Duke University Medical Center, Durham, NC, United States of America; 4 Department of Biostatistics and Bioinformatics, Duke University Medical Center, Durham, NC, United States of America; 5 Department of Pathology, Section on Comparative Medicine, Wake Forest School of Medicine, Winston-Salem, NC, United States of America; 6 Department of Medicine, Duke University School of Medicine, Durham, NC, United States of America; Northwestern University Feinberg School of Medicine, UNITED STATES

## Abstract

While exposure to radiation can be lifesaving in certain settings, it can also potentially result in long-lasting adverse effects, particularly to hematopoietic and immune cells. This study investigated hematopoietic recovery and immune function in rhesus macaques Cross-sectionally (at a single time point) 2 to 5 years after exposure to a single large dose (6.5 to 8.4 Gray) of total body radiation (TBI) derived from linear accelerator-derived photons (2 MeV, 80 cGy/minute) or Cobalt 60-derived gamma irradiation (60 cGy/min). Hematopoietic recovery was assessed through measurement of complete blood counts, lymphocyte subpopulation analysis, and thymus function assessment. Capacity to mount specific antibody responses against rabies, *Streptococcus pneumoniae*, and tetanus antigens was determined 2 years after TBI. Irradiated macaques showed increased white blood cells, decreased platelets, and decreased frequencies of peripheral blood T cells. Effects of prior radiation on production and export of new T cells by the thymus was dependent on age at the time of analysis, with evidence of interaction with radiation dose for CD8+ T cells. Irradiated and control animals mounted similar mean antibody responses to proteins from tetanus and rabies and to 10 of 11 serotype-specific pneumococcal polysaccharides. However, irradiated animals uniformly failed to make antibodies against polysaccharides from serotype 5 pneumococci, in contrast to the robust responses of non-irradiated controls. Trends toward decreased serum levels of anti-tetanus IgM and slower peak antibody responses to rabies were also observed. Taken together, these data show that dose-related changes in peripheral blood cells and immune responses to both novel and recall antigens can be detected 2 to 5 years after exposure to whole body radiation. Longer term follow-up data on this cohort and independent validation will be helpful to determine whether these changes persist or whether additional changes become evident with increasing time since radiation, particularly as animals begin to develop aging-related changes in immune function.

## Introduction

While radiation exposure can be lifesaving in the setting of cancer treatment or bone marrow transplantation for genetic or acquired disease, adverse effects may include cytopenias, abnormal immune function, delayed healing, fibrosis, and secondary malignancies, among others. The benefits of radiation are thought to outweigh the adverse effects for therapeutic radiation exposures. However, biologically significant non-beneficial radiation exposures may also potentially occur due to accidents, intentional non-therapeutic exposures (i. e. terrorist attacks), or prolonged space travel. Thus, it is important to assess the acute and long-term effects of such exposures to facilitate the development of medical countermeasures that can mitigate their harm. Abnormal function of the immune system is one of the adverse effects of radiation exposures that can potentially persist long-term (reviewed in [1]).

Immune responses depend on the presence and proper activity of both innate and adaptive immune cells. Innate immune cells include natural killer (NK) cells, dendritic cells, macrophages, and other myeloid cells that can recognize and immediately respond to a variety of markers (antigens). Adaptive immune cells include T and B cells that develop immune responses when their antigen-specific receptors bind to the corresponding antigens on potential pathogens. While B cells primarily respond to antigens by producing antibodies, T cells can respond either via producing cytokines that regulate immune responses or via direct cytotoxicity. As important regulators of both activation and resolution of immune responses, the numbers and activity of T cells are critical for proper defense against foreign invaders.

T cells develop from hematopoietic progenitor cells that migrate to the thymus, where they generate unique antigen receptors that are selected for appropriate reactivity with foreign antigens presented by other immune cells and the absence of reactivity against self-antigens. The thymus is largest and most active early in life as it establishes the initial peripheral T cell repertoire. Progressive age-related thymic involution occurs subsequently, although the thymus normally continues to produce a low number of T cells throughout the adult life [2–5]. The potentially novel antigen receptor specificities carried by these newly produced naïve T cells (thymic emigrants) have been hypothesized to be critical to maintain broad polyclonal immunity and to achieve full immune reconstitution without the development of “holes” in the immune repertoire following an insult such as chemotherapy or radiation that results in depletion of the previously established peripheral T cell repertoire [6,7]. Thymic function can be assessed non-invasively via direct detection in peripheral blood of phenotypically naïve T cells and/or extrachromosomal circles of DNA called signal joint T cell receptor excision circles (sjTRECs) that are excised during T cell receptor gene rearrangement in the thymus [8]. Since sjTRECs are not replicated during T cell proliferation in the periphery, their presence within cells provides a molecular indicator that those cells were produced *de novo* in the thymus.

The mechanisms of radiation-induced immune injury and acute recovery have been studied in a variety of small animal and primate models [9,10]. Studies that have focused on late effects of radiation (> 6 months post-radiation) have often shown incomplete hematopoietic and/or thymus recovery, especially in adults. For example, surgically post-menopausal “late-middle aged” female cynomolgus macaques (median age, 20 years; range 15–24 years) exposed to 5 Gray (Gy) radiation demonstrated acute, dose-dependent decreases in hematopoiesis that had not normalized by 6 months post-radiation. Thymus size and function were still decreased at 6 months for animals that had received either 2 or 5 Gy radiation [11]. Thymus tissues from humans exposed to up to 2.9 Gy radiation from the atomic bomb (A-bomb) in Hiroshima showed decreased thymic function at the time of their natural deaths 9–41 years later, compared to age-matched non-irradiated individuals [12]. Studies of how irradiation and thymic recovery affect immune responses to pathogens or vaccines generally have been limited by small cohorts, short follow-up times, and/or potential immune effects of underlying disease. Changes in circulating plasmacytoid dendritic cells were recently documented in 153 Japanese female atomic bomb survivors more than 60 years after radiation exposure [13] suggesting potential effects on immune function. Unfortunately, interpretation of some findings in the human A-bomb survivor studies are often complicated due to age related effects on the immune system. For example, a recent study of the effects of prior A-bomb radiation exposure on immune responses to influenza vaccine was limited by the overall poor antibody responses of both elderly control and irradiated subjects [14]. In addition, the studies in human A-bomb survivors are limited by variable radiation doses, heterogeneous exposures, differing ages at time of exposure, complications by under-nourishment, and retrospective analysis.

The rhesus macaque non-human primate (NHP) model has been shown to be an excellent model for study of immune system homeostasis and function [15] including studies that assess radiation countermeasures [11,16–18]. We used this model to test the hypothesis that previous irradiation would impair hematopoiesis and/or humoral and cell-mediated immune responses years after irradiation, using both phenotypic and functional analyses. Hematopoietic recovery was assessed in a large cohort of rhesus macaques at a median of ~5 years post-acute radiation exposure. The capacity of a subset of these animals to mount antibody responses was determined by challenge with rabies, *Streptococcus pneumoniae*, and tetanus toxoid antigens. Individual variation in immune recovery was assessed through measurement of complete blood counts, lymphocyte subpopulation analysis, and thymus function (via signal joint T cell receptor excision circles, sjTRECs) in the larger cohort and by post-vaccine response antibody titers in the antigen-challenged subset.

## Materials and methods

### Subjects studied

A total of 43 male rhesus macaques (*Macaca mulatta*; 29 irradiated and 14 non-irradiated controls) were studied. All *in vivo* blood collections, immune challenges, and other post-irradiation procedures were conducted at the Wake Forest University School of Medicine with approval by the Institutional Animal Care and Use Committee of Wake Forest University. Wake Forest University is committed to provide a high-quality program of animal care in compliance with state and federal Animal Welfare Acts and the standards and policies of the US Department of Health and Human Services. Wake Forest University has an Assurance on file in the Office for Protection from Research Risks, Office of the Director, National Institutes of Health, that accepts responsibility for the humane care and use of animals (OPRR #A-3391-01). The Laboratory Animal Care Program of the Wake Forest University School of Medicine complies with the "Principles for Use of Animals", the "Guide for the Care and Use of Laboratory Animals" [19], all provisions of the Animal Welfare Act, and has been accredited by the Association for Assessment and Accreditation of Laboratory Animal Care, International (AAALAC) since April 8, 1966 (AAALAC File #8).

The control and irradiated animals used for this study were obtained from Wake Forest University, University of Maryland, University of Illinois, Armed Forces Radiobiology Research Institute, and Primate Products. Irradiated animals received 6.5 to 8.4 Gy total body radiation under IACUC oversight at their prior institution using one of two strategies: (1) linear accelerator-derived photons at a nominal mean energy of 2 MeV, delivered at 80 cGy/minute as a split dose given half anterior-posterior and half posterior-anterior; or (2) Cobalt 60-derived gamma irradiation delivered simultaneously, bilaterally at 60 cGy/min. Note that these are potentially lethal doses: the LD 10/30 for rhesus macaques is ~5.5 Gy, the LD 50/30 is ~6.7 Gy, and the LD 90/30 is 8 Gy [20]. Surviving animals were subsequently transferred to Wake Forest School of Medicine Center for Comparative Medicine Research for long-term monitoring post-radiation. Irradiation methods, supportive care strategies, and acute effects for many animals donated to this cohort have been recently reported [17, 20, 21].

After arrival at Wake Forest, all animals were monitored twice daily by trained veterinary technical laboratory staff to assure animal well-being and social stability. Any evidence of illness was classified using a nonhuman primate-specific modification of the Children’s Clinical Oncology Group toxicity criteria [22]. Sick animals were promptly evaluated by one of seven institutional veterinarians independent of the research team, two of whom are board-certified by the American College of Laboratory Animal Medicine.

Animals consumed a diet of laboratory chow (Purina LabDiet Monkey Diet 5038, Richmond, IN, USA) that was supplemented with fresh fruits and vegetables and with water *ad libitum*. They were housed socially in indoor-outdoor pens whenever possible, or in group cages if necessary for safe handling or medical care. Care was taken to ensure the animals in the groups were compatible. Environmental enrichment, including fruits/vegetables, toys, puzzles, climbing and hiding environments, was provided continuously on a rotating basis. Behavioral well-being was additionally monitored and recommendations made as needed by an independent behavioral management team. All animals were trained to cooperate in handling procedures, to minimize stress. Sampling was scheduled so that the animals were sedated the minimum number of times required for data collection.

**Fig 1:**
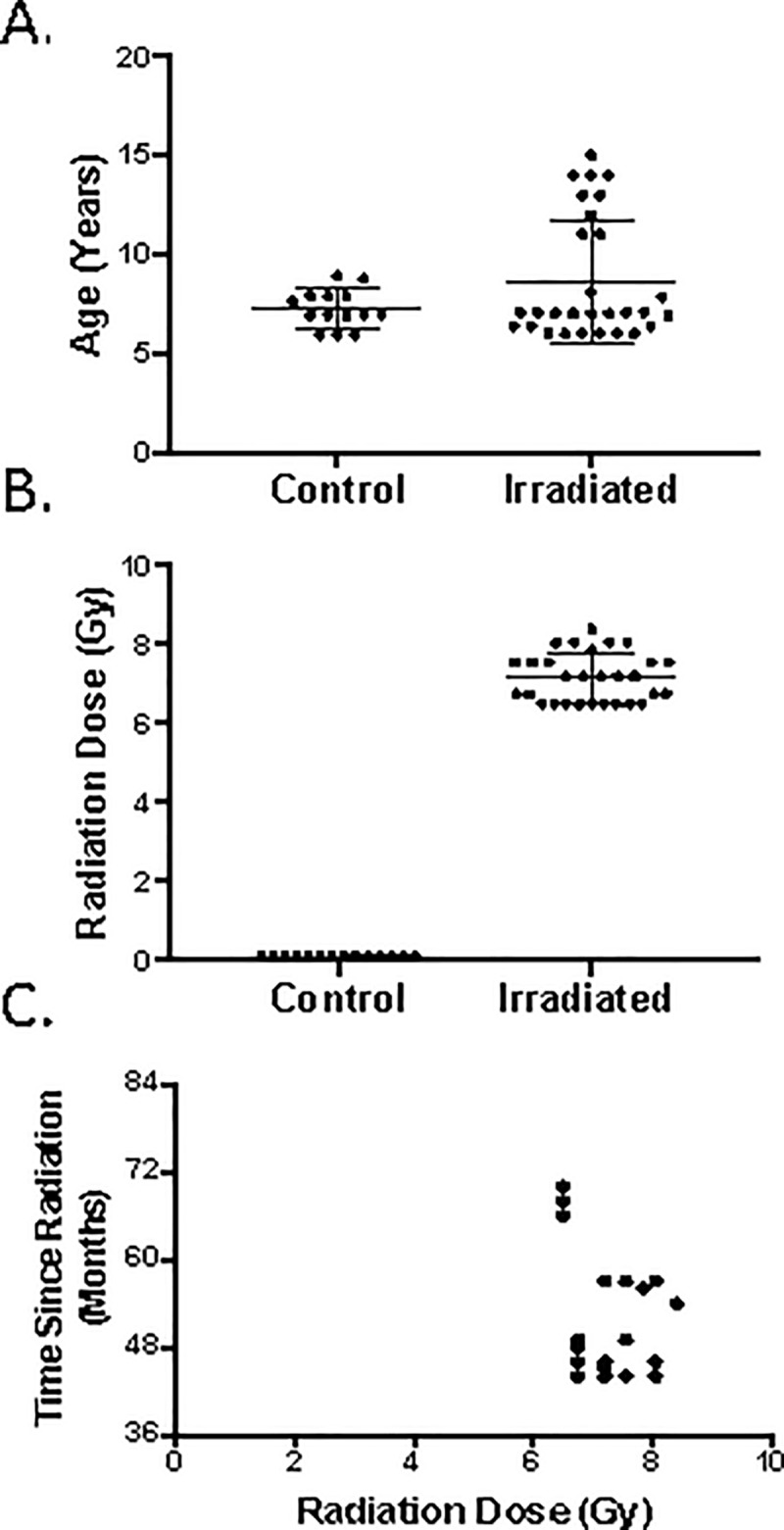
Demographics of the non-human primate population studied. **A**. The ages are shown for control (n = 14) and irradiated animals (n = 29) in the X-10-12 cohort. The median age of 7 years was similar for both groups, with a range from 5.9 to 8.9 years for the control group and a range of 6 to15 years for the irradiated group. **B**. The median dose (Gy) received by irradiated animals was 7.2 Gy, with a range of 6.5 to 8.4 Gy. **C.** The median time between radiation exposure and study/blood collection was 55 months, with a range from 44 to 70 months.

Characteristics of the population studied are summarized in Fig 1. The median age of the animals was seven years at the time of this study, corresponding to early adulthood [15], with a range of 5.9 to 8.9 years in the non-irradiated control group and 6 to 15 years in irradiated animals (Fig 1A). The median dose received by irradiated animals was 7.2 Gy (Fig 1B). The hematologic evaluation was performed at a median of 55 months (range 44–79 months) post irradiation (Fig 1C).

### Hematologic and immunologic recovery monitoring

To assess hematologic and immunologic recovery post-radiation, animals were sedated with intramuscular ketamine and blood was drawn from the femoral vein for complete blood counts (CBC) including hematocrit, red blood cells, red blood cell indices, total and differential leukocyte counts, hemoglobin, and platelet count. Lymphocyte phenotype was determined by flow cytometry on peripheral blood mononuclear cells derived by density gradient centrifugation of heparinized blood as described [15,23,24]. Analysis of sjTRECs as an indicator of thymus function was performed on bead-isolated CD3^+^ cells derived from peripheral blood as previously described [8].

### Vaccination response monitoring in a subset of the cohort

Nine young adult male rhesus macaques that had previously been irradiated (August 2006) with 7.5 to 8.5 Gy of 6 mega volt (MV) photons at the University of Maryland and four non-irradiated control animals participated in a sub-study to assess immune recovery post-irradiation. Mean age at the time of irradiation was 3.7 years (range 3.1–4.4 years). All 13 animals were immunized 2 years later on day 0 (July 2008) with a single dose of the polyvalent *Streptococcus pneumoniae* vaccine PneumoVax 23 (Merck Sharp and Dohme Corp., Kenilworth, NJ), on day 31 with the rabies vaccine Defensor 1 (Pfizer Animal Health, New York, NY), and on day 60 with a tetanus toxoid vaccine (Sanofi Pasteur, Swiftwater, PA). Age at initiation of the vaccine challenge was 5.6 years [range 5.0–6.3] in irradiated subjects and 5.2 years [range 4.2–6.2] in controls. Peripheral blood (≤ 7 mL/draw) was collected at baseline and at 1, 4, 8, and 16 weeks following each respective immunization for assessment of antibody titers against the vaccines.

### Multiplex Luminex pneumococcal (Pnc) anti-capsular IgG capture assay

IgG antibodies directed against the capsule of pneumococcus serotypes 1, 3, 4, 5, 6B, 7F, 9V, 14, 18C, 19F, and 23F were quantitated using a customized multiplex (11-plex) pneumococcal (Pnc) anti-capsular IgG capture assay performed by the Centers for Disease Control and Prevention (CDC) based on previous methods with modifications [25]. Briefly, Pnc serotype-specific capsular polysaccharides (Ps) (American Type Culture Collection, Manassas, VA) were conjugated to poly-L-lysine (PLL, Sigma, St. Louis, MO) [26,27] and then to carboxylate-modified microspheres (Luminex Corp, Austin, TX) using a two-step carbodiimide reaction [28]. PLL-Ps beads for 11 Pnc serotypes (5,000 beads/serotype in 25 μL volume) and serum samples (25 μL /well) were transferred to a 96-well multi-screen filter plate and incubated at 37°C for 30 minutes with 150 rpm agitation. Plates were washed three times with wash buffer (PBS containing 0.05% Tween 20 and 0.2% newborn bovine serum albumin (NBBS, Sigma, St. Louis, MO). Fifty μL/well of R-phycoerythrin (R-PE)-conjugated anti-monkey IgG (Southern Biotech, Birmingham, AL) was added and incubated at 37°C for 30 minutes with 150 rpm agitation. Plates were washed three times in wash buffer, beads re-suspended in 130 μL wash buffer /well, and data was acquired in a Luminex reader (Luminex 200, Luminex Corp, Austin, TX) with Masterplex CT software suite (MiraiBio, South San Francisco, CA). Results reported represent the mean of three independent runs by two different operators, reported in arbitrary units relative to reference serum RS2010 as described below. A response to vaccination was defined as an increase in serotype-specific IgG from baseline (day 0) that persisted to 16 weeks post-vaccination.

Four sera (C1655, C1484, R1579 and R1580) with high signals for Pnc serotype (ST)- specific IgG were combined to generate the rhesus reference standard, RS2010. A RS2010 dilution curve was generated by plotting median fluorescence intensity (MFI) against dilution. Pnc ST-specific IgG values for each ST in RS2010 were calculated in arbitrary units (AU) = MFI × dilution factor. RS2010 was included for 8 dilutions in duplicate on each assay plate and the Pnc ST-specific IgG values in AU/mL were computed for each sample from the RS2010 standard curve using Masterplex QT software (MiraiBio, South San Francisco, CA) (S1 Fig).

### Antibody responses to tetanus toxoid and rabies

Commercially available enzyme-linked immunosorbent assays (ELISAs) were used to assess IgG and IgM responses to tetanus toxoid (Monkey Anti-Tetanus Toxoid IgG and IgM ELISA Kits, Life Diagnostics, Inc.) and rabies (Platelia Rabies II Kit, BIO-RAD, Steenvoort France). Assays were run according to manufacturer’s instructions.

### Statistical analysis

The Jonckheere-Terpstra test [29,30] was used to test for association of hematologic parameters with prior radiation dose. The Wilcoxon test [31,32] was used to test association between pre-radiation and hematologic parameters. Kendall’s test [33] was used to test the association of age with hematologic parameters. A linear regression model was to used to estimate the interaction of age and dose with respect to hematologic parameters. Differences of immune responses to vaccination between irradiated and control animals were determined using Fisher’s test [34,35]. The analyses have not been adjusted for multiple testing. All statistical analyses were carried out using the R statistical enviroment [36] along with extension packages coin [37] and clinfun [38]. Packages from the tidyverse [39] ecosystem and the knitr [40] extension package were used to facilitate adherence to the principles of reproducible analysis.

## Results

### Total body irradiation induced persistent changes in hematologic parameters and circulating lymphocyte subsets

**Fig 2:**
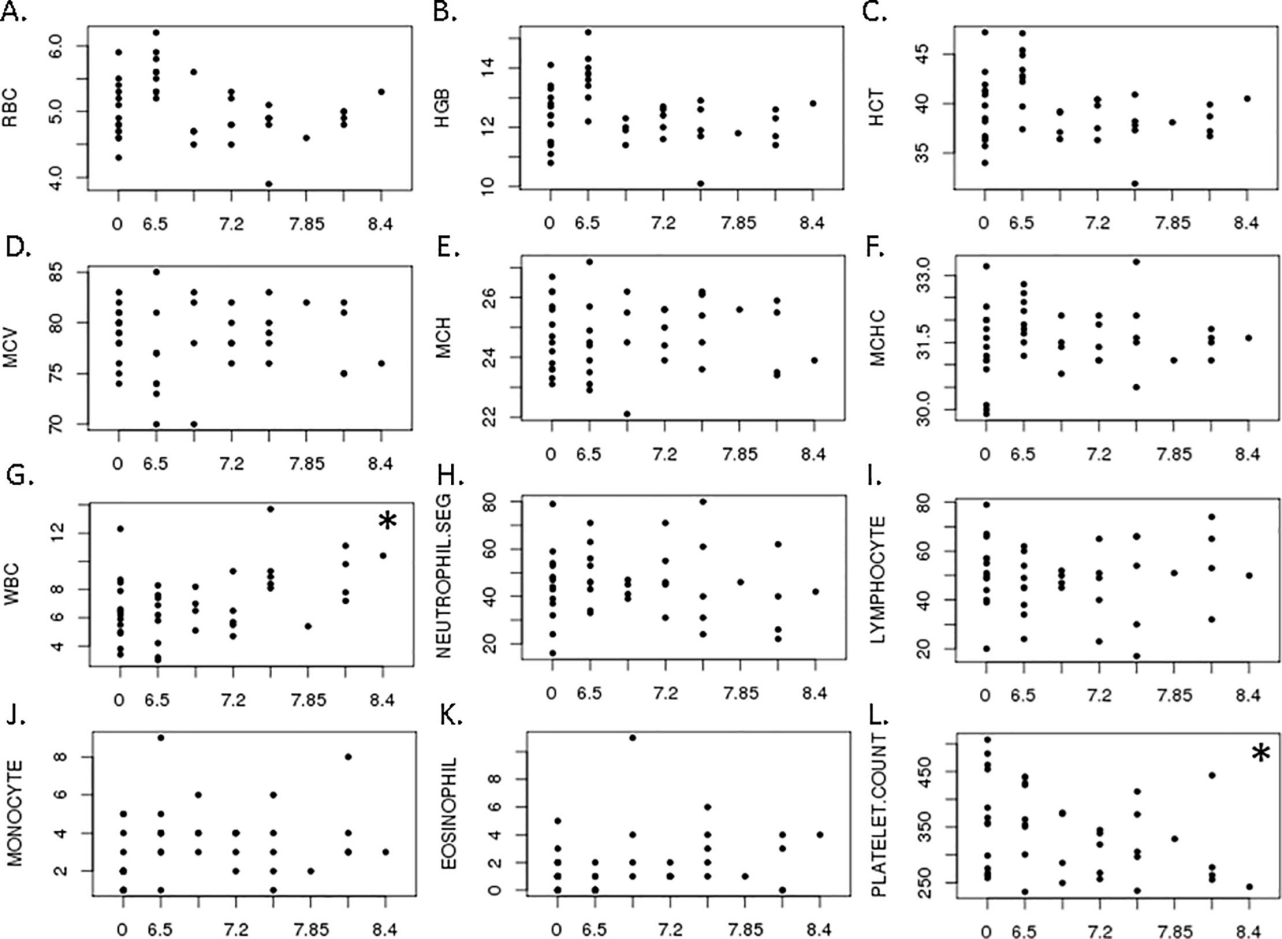
Hematologic parameters at a median of 55 months (~ 5 years) post-irradiation. Values of each parameter are shown as a function of radiation dose in Gy (x-axis for all plots); 0 Gy represents the control group. Each point represents a different subject (n = 43). **A**. Red blood cell (RBC) concentration, cells x 10^6^/μL; **B**. Hemoglobin (Hgb), g/dL; **C**. Hematocrit (Hct), %; **D**. Mean corpuscular volume (MCV), fL; **E**. Mean cellular hemoglobin, (MCH), pg; **F**. Mean cellular hemoglobin concentration (MCHC), g/dL; **G**. White blood cells (WBC), cells x 10^3^/μL; **H**. Neutrophil %; **I**. Lymphocyte %; **J**. Monocyte %; **K**. Eosinophil %; **L**. Platelet count, x 10^3^/μL. Asterisk (*) indicates p = 0.01 in panel G and p = 0.05 in panel L for dose-based comparison of irradiated and control animals using the Jonckheere-Terpstra test. Raw data are provided in S1 Table.

At a median of 55 months post- radiation, previously irradiated animals (n = 29) had similar red blood cell counts, hemoglobin, hematocrit, red blood cell indices, and percentages of whole blood neutrophils, lymphocytes, monocytes, eosinophils, and basophils as control animals (n = 14) (Fig 2A–2K). However, total white blood cell (WBC) counts in whole blood at this cross-sectional sample increased (Fig 2G; p = 0.01) and platelet counts decreased as radiation dose increased (Fig 2L; p = 0.05). A linear regression analysis indicated that these relationships were not adequately described using a linear model (p = 0.08 and 0.13, respectively). As an example of the differences observed, total WBC counts were 6.5 ± 0.6 x 10^3^/μL (mean ± SEM; n = 14) in control animals and 9.7 ± 1.0 x 10^3^/μL (n = 5) in animals that received 7.55 Gy. Similarly, platelet counts were 357 ± 24 x 10^3^/μL (mean ± SEM; n = 14) and 325 ± 31 x 10^3^/μL (n = 5) in control animals versus those that received 7.55 Gy, respectively.

**Fig 3:**
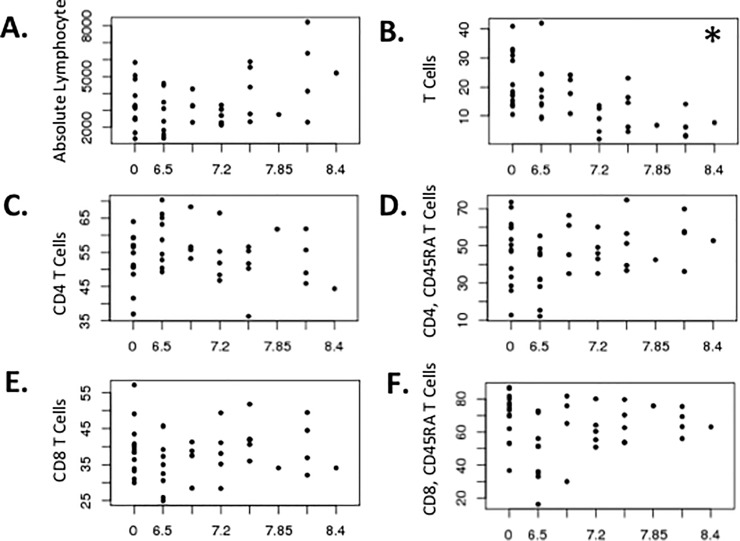
Lymphocyte subset recovery at a median of 55 months (~ 5 years) post-irradiation. Values of each parameter as determined by complete blood count (**A**) or flow cytometry of peripheral blood mononuclear cells isolated by density gradient centrifugation (**B–F**) are shown as a function of radiation dose in Gy as the x-axis for all plots. Each point represents a different subject (n = 43). **A**. Absolute lymphocyte counts, cells x 10^6^/μL; **B**. T cell %; **C**. CD4 T cell %; **D**. CD4 CD45RA^high^ (naïve) %; **E**. CD8 T cell %; **F**. CD4 CD45RA^high^ (naïve) %. * indicates Asterisk (*) in panel B indicates p = 0.0004 for dose-based comparison of irradiated and control animals using the Jonckheere-Terpstra test. Raw data are provided in S1 Table.

To further assess the effects of radiation, peripheral blood mononuclear cells isolated from heparinized blood were immunophenotyped with flow cytometry. Although absolute lymphocyte counts obtained from whole blood did not differ (Fig 3A), the percentage or frequency of T lymphocytes in the isolated peripheral blood mononuclear cells were decreased with radiation dose (Fig 3B; p = 0. 0004). A linear regression analysis showed that the decrease in T lymphocyte frequency was well-described by a linear model (p = 0.0006), with a slope of -1.3%/Gy. As expected, naïve T cells, indicated by high surface expression of the CD45RA marker (CD45RA-high), were higher in younger animals (p = 0.002 for CD4+ CD45RA-high cells and p = 0.02 for CD8+ CD45RA-high cells). Although irradiated and control animals had similar percentages of CD4+ versus CD8+ T cell subsets and fractions of these cells that were naïve (CD45RA-high) (Fig 3C–3F), linear regression analysis showed that the numbers of naïve CD8+ T cells depended on age (p = 0.01) and radiation dose (p = 0.04), with suggestion of an interaction (p = 0.05) between age and prior radiation dose.

**Fig 4:**
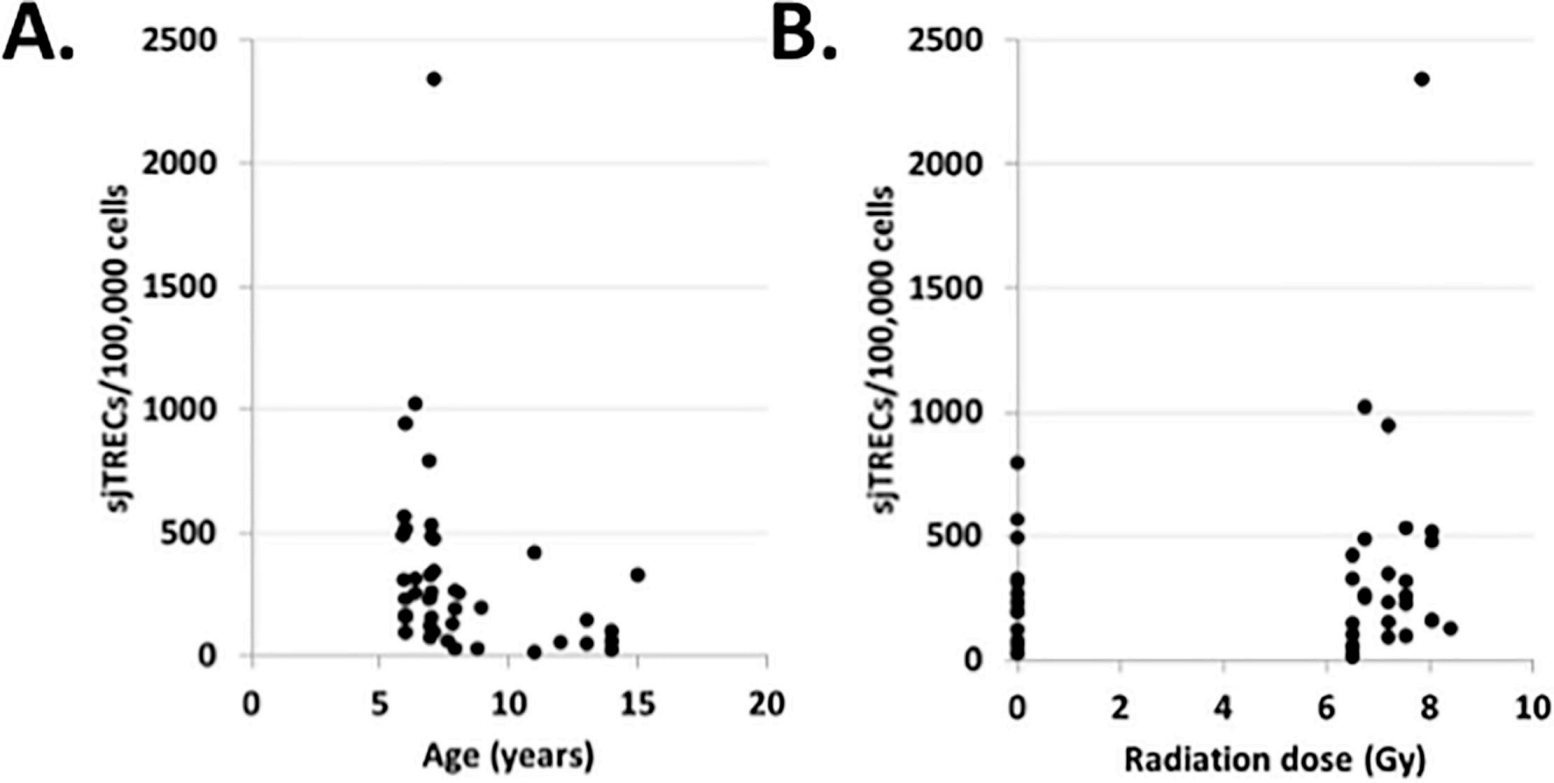
Thymus function ~ 5 years post-irradiation. sjTREC levels in isolated CD3^+^ mononuclear cells from peripheral blood are plotted as a function of age (**A**) and radiation dose (**B**). Each point represents a different subject (n = 43). In panel A, the open triangles show values for control, non-irradiated animals and the solid black circles show values for irradiated animals. sjTRECs were associated with age (p = 0.002), but did not vary according to radiation dose. Raw data are provided in S1 Table.

sjTREC analysis was used to further assess the late effects of prior radiation on thymus function. The number of sjTRECs, a molecular marker for T cells that recently emigrated from the thymus, were significantly higher in younger animals (Fig 4A; p = 0. 0.002) but did not differ significantly based on the radiation dose (Fig 4B).

### Antibody responses to polysaccharide antigens remain compromised ~2 years post-total body irradiation

To test the hypothesis that prior irradiation leads to lasting effects on immune function, nine previously irradiated and four control macaques were immunized with commercially available vaccines against rabies, *Streptococcus pneumoniae*, and tetanus toxoid and antibody responses were determined. The proportion of animals that responded to polysaccharide antigens were similar for control and irradiated animals for 10 of the 11 pneumococcal serotypes tested (Table 1). There was also no difference in the number of serotypes to which individual macaques responded, ranging from 4 to 10 of 11 antigens tested in the control group and from 0 to 9 of 11 antigens tested in the irradiated group (p = 0.53). However, while 4/4 (100%) of control macaques mounted antibody responses to polysaccharide antigens from serotype 5 pneumococcus, 0/9 (0%) of irradiated animals responded to this serotype (p = 0. 001). Taken together, these results suggest that immune responses to certain polysaccharide antigens may be compromised in previously irradiated animals, leading to a “hole” in their immune repertoire for the serotype 5 polysaccharide and perhaps other antigens.

**Table 1:** Antibody responses to pneumococcal (Pnc) vaccine ~2 years post-irradiation.

	PnC Serotypes Tested											
**Animal ID**	**1**	**3**	**4**	**5**	**6B**	**7F**	**9V**	**14**	**18C**	**19F**	**23F**	**Total** **Positive**
C1	++	++	-	++	-	++	-	++	-	-	++	**6/11**
C2	++	++	++	++	++	++	++	++	++	++	-	**10/11**
C3	++	++	-	++	-	++	++	++	++	++	-	**8/11**
C4	-	++	-	++	-	-	-	-	++	++	-	**4/11**
**Control (%+)**	**75%**	**100%**	**25%**	**100%**	**25%**	**75%**	**50%**	**75%**	**75%**	**75%**	**25%**	
R1	++	++	++	-	++	-	-	++	++	-	++	**7/11**
R2	-	-	-	-	-	-	-	-	-	-	-	**0/11**
R3	++	++	++	-	-	++	-	++	++	++	++	**8/11**
R4	-	++	++	-	-	-	-	-	++	-	-	**3/11**
R5	-	++	++	-	-	++	++	++	++	++	++	**8/11**
R6	-	++	-	-	-	-	-	++	++	++	++	**5/11**
R7	++	++	++	-	++	-	++	++	++	++	++	**9/11**
R8	++	++	-	-	-	++	-	-	-	-	-	**3/11**
R9	-	++	-	-	-	++	++	-	++	-	++	**5/11**
**Radiated (%+)**	**44%**	**89%**	**56%**	0%*	**22%**	**44%**	**33%**	**56%**	**78%**	**44%**	**67%**	

Note: A positive response (++) was classified based on a sustained increase in IgG response lasting to 16 weeks after vaccination, compared with day 0 (see S2 Fig). Lack of a positive response is indicated by (-).

* indicates a significant difference in response between control and previously irradiated animals (p = 0.001).

### Mean antibody responses to protein antigens have generally normalized by ~2 years post-total body irradiation

**Fig 5:**
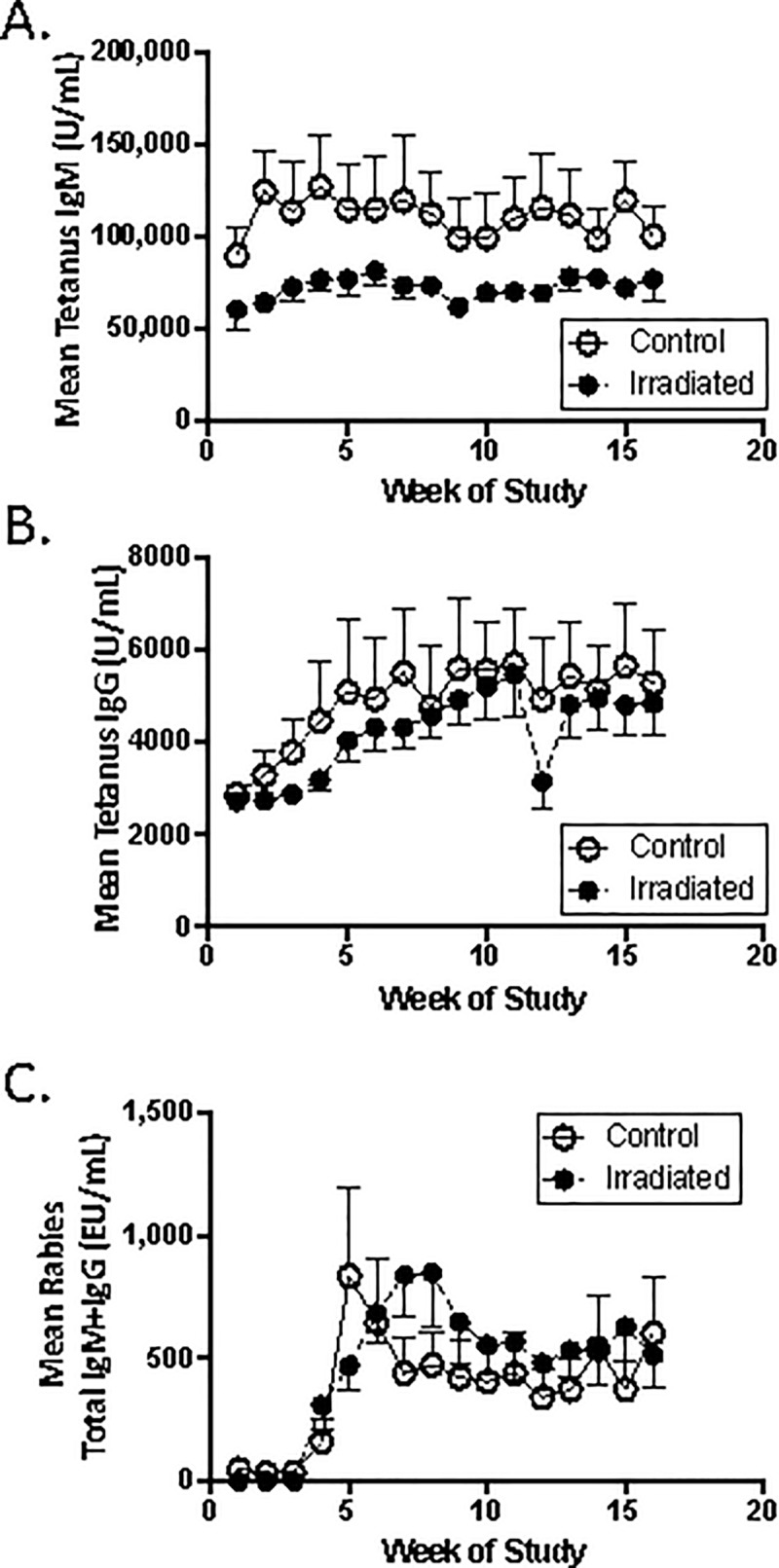
Antibody responses to tetanus and rabies antigens ~ 2 years post-irradiation. **A**. IgM responses to tetanus vaccine. **B**. IgG responses to tetanus vaccine. **C**. Combined IgM/IgG responses to rabies vaccine. Raw data are provided in S2 Table.

Mean IgM and IgG responses to tetanus toxoid and mean rabies combined IgM/IgG responses were statistically similar in irradiated versus control animals (Fig 5). However, irradiated animals demonstrated a strong, but non-statistically significant, trend toward decreased serum anti-tetanus IgM at all time points studied (Fig 5A). A non-significant trend toward a delay in mean peak antibody responses to rabies vaccine was also seen in irradiated animals compared with controls (Fig 5C). Larger studies with increased statistical power will be required to determine whether these trends reflect residual impairments in antibody production in irradiated animals.

Interestingly, one seven year old macaque who had received 7.85 Gy radiation prior to vaccination failed to make antibodies against 6/11 pneumococcal serotypes tested (R2 in Table 1), and had only transient responses to 5 antigens which had disappeared by 16 weeks post-vaccination. At 56 months post-irradiation, this animal had an absolute lymphocyte count of 2,754 cells/μL with 7% T cells and was an outlier with regard to sjTRECs (2,340/10^5^ CD3^+^ cells). His IgM responses to tetanus were lower than the mean for irradiated animals, IgG responses to tetanus were similar to the mean, and his combined IgG/IgM responses to rabies vaccine were similar to or above the mean, indicating that this animal had isolated deficient responses to polysaccharide but not protein antigens.

## Discussion

This study of hematopoietic recovery and immune function several years after radiation exposure shows that the irradiated macaques experienced dose-related thrombocytopenia, increased total white blood cell counts, and decreased percentages of T cells in peripheral blood, as a prolonged or delayed effect of radiation exposure. Effects of prior radiation on production and export of new T cells by the thymus gland was associated with age, with evidence of interaction of age and radiation exposure dose for CD8+ T cells. Irradiated and control animals generally developed similar humoral responses to protein antigens and most of the polysaccharide antigens tested, although irradiated animals uniformly failed to make antibodies against polysaccharides from serotype 5 pneumococci. Trends toward decreased serum IgM responses to tetanus and slower peak antibody responses to rabies were also observed, which need to be confirmed by larger studies with increased statistical power. Taken together, this study suggests that while irradiated animals respond to most antigens, minor impairment of immune responses persisted in these relatively young subjects despite ~ 2 years of recovery, particularly relative to polysaccharide antigens.

Irradiated macaques in the larger, broader age range cohort demonstrated long-standing decreases in platelet counts as well as dose-related increases in total peripheral blood white blood cell counts relative to control animals. Prior radiation exposure has been documented to increase peripheral white blood cell counts and chronic inflammation, as demonstrated by increased erythrocyte sedimentation rates in human survivors of atomic bomb blasts [41]. These increases in inflammation have been documented to occur via a variety of mechanisms, including direct damage to cells, cellular stress responses, endovascular injury that results in vascular leak, excessive activation of M1 pro-inflammatory macrophages, and the senescence-associated secretory phenotype (reviewed in [42]). The relative contributions of any of these specific mechanisms to the differences seen in this study are unknown. In addition, since these animals were studied cross-sectionally and were heterogeneous with respect to age, age at irradiation, time since irradiation, and radiation dose, additional follow-up studies will be required to confirm whether the observed differences are representative across populations.

Irradiated macaques demonstrated dose-related decreases in the percentages of T cells present in peripheral blood. Since T cells are generated in the thymus, we investigated several markers that can provide insight into thymus gland function (i. e., thymopoiesis). The data presented here show that levels of sjTRECs were highest in younger animals and were not associated with radiation dose. The generation of new naïve T cells in the thymus depends both on availability of precursor cells that migrate from the bone marrow and thymus-intrinsic factors that affect the output of T cells. Effects of either radiation or aging on thymopoiesis are multi-factorial and depend on survival and repopulation of bone marrow hematopoietic stem cells (HSC), potential radiation-induced skewing of functional responses with respect to cell lineage repopulation properties, migration to the thymus, appropriate seeding of thymic niches, and completion of the thymocyte/T cell maturation processes. The relative contributions of each of these factors is still poorly understood. However, the data suggest that animals irradiated early in life are more resilient for recovery of thymopoiesis, compared to those irradiated later in life.

Consistent with this, increased percentages of both CD4+ and CD8+ naïve cells detected by flow cytometric immunophenotyping were also significantly associated with younger animals. However, linear regression analysis also suggested that a significant interaction between age and prior radiation dose was limited to the CD8+ naïve T cell population. It is important to note that our analysis was based on age at the time of blood draw, and that animals were irradiated at ages of 5.9 to 15 years and their thymus function was assessed 44 to 70 months later. Also, while T cell percentages varied by dose, it is very difficult to separate variability associated with the dose range used (6.6 to 8.4 Gy) given the relatively small number of animals assessed and assay variability. The animals were exposed to radiation at different research sites that also confounds dose delivery to target sites, relative dosimetry etc. Additional studies may be necessary to fully address these issues in more controlled settings.

Previous work in ovariectomized female cynomolgus macaques showed that total body irradiation sufficient to cause acute hematopoietic syndrome (e.g. 2–5 Gy) resulted in decreased thymus weight with marked atrophy of thymic cortex and medulla and decreased sjTRECs that remained evident at least 24 weeks post-irradiation [11]. The data presented here using a broader age range of male rhesus macaques showed that age had a stronger influence on the production and export of new T cells by the thymus than total body irradiation doses when assessed ~5 years post-irradiation, although there was evidence of significant interaction with radiation dose for CD8+ T cells (p = 0.05). At first glance, these results may seem discordant with prior work that demonstrated significantly decreased long-term thymic function in humans exposed to atomic bomb radiation [12] and in experimentally irradiated mice [43]. However, the studies presented here differed in several ways from those previously published, including age at irradiation, radiation doses, the time elapsed between radiation and assessment relative to the lifespan of the organism, and the methods used to assess thymus function. The thymus of young mice has been shown to rapidly regenerate in weight and production of T cells following acute radiation exposure (reviewed in [43]). However, thymic regeneration is slower in older mice and humans and may not reach pre-irradiation baseline levels (reviewed in [44]). The available data in humans suggests that radiation exposure may not necessarily prevent full thymus regeneration acutely, but may accelerate age-related thymus involution [12]. Thus, the full effects of radiation on the thymus may not yet be evident in the cohort of macaques studied, where most animals were irradiated in “late adolescence” and studied as young adults, when thymic reserve is still substantial.

Although antibody responses to most of the administered vaccine antigens were statistically similar in irradiated and control animals, one exception emerged–the response to the polysaccharide antigens from pneumococcus serotype 5 –where 4 of 4 control animals produced antibodies versus 0 of 9 irradiated animals. Immune responses to polysaccharides require a more developed immune system, such that strong responses to these antigens are not typically seen in humans until about two years of age. The “hole in the immune repertoire” toward the serotype 5 polysaccharides demonstrated by irradiated animals (p = 0.001) may thus reflect subtle radiation-associated decreases in immune function. This interpretation is supported by the non-significant trend toward decreased IgM levels produced against tetanus toxoid, the delayed peak antibody responses to rabies that were also observed in irradiated animals, and the observation that one irradiated macaque who responded to the vaccine protein antigens had only transient responses to 5 of the polysaccharide antigens and this response had disappeared by the 16 week timepoint. Interestingly, the polysaccharide expressed by serotype 5 pneumococcus is unique among the strains contained in the 23-valent pneumococcal vaccine, by the replacement of glucose in the repeating unit of its capsular polysaccharide with galactose [45,46]. How this structural variation might influence immunogenicity in irradiated animals is unclear, and whether skewing of the recovery of thymopoiesis relative to recovery of other hematopoietic cell types contributes to this observation is unknown. It is also unknown whether the antibodies made by irradiated animals differ in their functional (opsonic) activity and thus their ability to protect against disease compared to control animals. Importantly, radiation-associated immune deficits may become more pronounced as the animals age, since aging has independently been associated with decreased thymic T cell production, frequent clonal expansion of memory T cell populations, increases in autoimmune diseases/syndromes, and diminished response to vaccinations [47–50].

In summary, the data presented show that dose-related changes in peripheral blood cells and in immune responses to antigens were detected at 2 to at least 5 years after exposure to radiation. Additional studies and longer term follow-up research is needed to determine whether these changes persist and the impact of the alterations on other systems of the body. These data are relevant to the question of whether pathologies become more evident with increasing time since radiation, particularly as subjects begin to develop aging-related deficits in immune function.

## Supporting information

S1 Table Hematologic and immunologic parameters in peripheral blood of irradiated and control macaques (raw data). The units used for each measurement are provided in Figs 2–4.
(PDF)

S2 Table Vaccine responses against tetanus and rabies (raw data). (PDF)

S1 Fig Assignment of standard values using reference serum RS2010. Reactivity of RS2010 with each pneumococcal serotype polysaccharide was used to define IgG responses to that serotype in arbitrary units (AU), defined as median fluorescence intensity in the Luminex-based immunoassay x dilution factor, as shown here for serotype 4.
(TIF)

S2 Fig Vaccine responses against pneumococcal serotypes. The mean IgG level and standard error of the mean (n = 3 replicates) is plotted in arbitrary units for each animal relative to reference serum RS2010 for each of 11 pneumococcal serotypes, as described in the Materials and Methods. To be considered a positive reaction to vaccination, the IgG level measured at week 16 post-vaccination had to be significantly increased relative to the IgG level measured at week 0, prior to vaccination. Designation of each response as positive (+) or negative (-) is indicated for each animal and serotype combination. **A**. Serotype 1. **B**. Serotype 3. **C**. Serotype 4. **D**. Serotype 5. **E**. Serotype 6B. **F**. Serotype 7F. **G**. Serotype 9V. **H**. Serotype 14. **I.** Serotype 18C. **J**. Serotype 19F. **K**. Serotype 23F. These results were summarized in Table 1 of the manuscript.
(PDF)
