# Supplementary material for: Late effects of total body irradiation on hematopoietic recovery and immune function in rhesus macaques
Source: PLoS One. 2019 Feb 13;14(2):e0210663. doi: 10.1371/journal.pone.0210663 (PMC6373904; doi:10.1371/journal.pone.0210663)
Supplement: S1 Table — The units used for each measurement are provided in Figs 2–4. (PDF) [file pone.0210663.s001.pdf]

| Code # | Age | DOSE (Gy) | MO. SINCE IRRAD | WBC | RBC | HGB | HCT | MCV | MCH | MCHC | NEUTR % | LYMPH% | MONO% | EOS% | PLATELETs | sjTREC/10e5 cells | ABSOLUTE LYMPHs | % T Cells | % CD4 T | % CD4 /CD45RA Hi T | % CD8 T | % CD8/CD45RA Hi T |
|---|---|---|---|---|---|---|---|---|---|---|---|---|---|---|---|---|---|---|---|---|---|---|
| C1 | 8.77 | 0 | | 7.9 | 5.9 | 14.1 | 47.2 | 80.0 | 23.8 | 29.9 | 44 | 49 | 5 | 2 | 264 | 25.2 | 3871 | 17.4 | 59.3 | 12.8 | 33.9 | 36.7 |
| C2 | 7.61 | 0 | | 3.4 | 5.4 | 12.8 | 41.2 | 76.0 | 23.6 | 31.1 | 54 | 39 | 5 | 2 | 358 | 54.8 | 1326 | 14.2 | 50.9 | 28.5 | 40.8 | 69.7 |
| C3 | 6.9 | 0 | | 6.4 | 4.8 | 11.1 | 35.7 | 74.0 | 23.1 | 31.1 | 16 | 79 | 2 | 3 | 276 | 792.0 | 5056 | 33.0 | 37.0 | 25.9 | 57.1 | 73.2 |
| C4 | 5.9 | 0 | | 8.5 | 4.9 | 11.5 | 36.7 | 75.0 | 23.6 | 31.4 | 39 | 57 | 3 | 1 | 268 | 490.0 | 4845 | 17.1 | 50.8 | 53.4 | 40.0 | 76.3 |
| C5 | 6.93 | 0 | | 6.2 | 4.6 | 12.1 | 36.3 | 79.0 | 26.2 | 33.2 | 59 | 40 | 1 | 0 | 356 | 326.0 | 2480 | 10.5 | 57.1 | 50.5 | 36.4 | 86.9 |
| C6 | 6.93 | 0 | | 5.5 | 4.7 | 12.4 | 38.5 | 81.0 | 26.2 | 32.3 | 37 | 57 | 2 | 2 | 264 | 74.0 | 3135 | 32.4 | 57.0 | 73.5 | 39.3 | 80.1 |
| C7 | 5.93 | 0 | | 5.9 | 5.2 | 13.4 | 41.9 | 80.0 | 25.7 | 32.0 | 43 | 55 | 1 | 1 | 367 | 308.0 | 3245 | 29.1 | 56.6 | 61.6 | 38.4 | 77.3 |
| C8 | 6.93 | 0 | | 12.3 | 5.5 | 13.3 | 43.2 | 78.0 | 24.2 | 30.9 | 79 | 20 | 1 | 0 | 462 | 118.4 | 2460 | 15.4 | 59.3 | 47.0 | 33.3 | 81.6 |
| C9 | 7.93 | 0 | | 3.8 | 5.1 | 13.0 | 40.9 | 81.0 | 25.6 | 31.8 | 53 | 44 | 2 | 1 | 385 | 264.0 | 1672 | 20.8 | 51.3 | 59.7 | 40.4 | 74.5 |
| C10 | 6.93 | 0 | | 4.9 | 4.3 | 10.8 | 34.0 | 80.0 | 25.1 | 31.6 | 32 | 66 | 2 | 0 | 259 | 232.0 | 3234 | 30.9 | 50.6 | 70.7 | 43.5 | 86.4 |
| C11 | 5.93 | 0 | | 6.5 | 4.7 | 11.5 | 38.2 | 82.0 | 24.5 | 30.1 | 48 | 49 | 2 | 1 | 454 | 562.0 | 3185 | 13.4 | 48.6 | 60.2 | 30.0 | 70.2 |
| C12 | 7.93 | 0 | | 6.6 | 5.3 | 12.4 | 41.3 | 78.0 | 23.3 | 30.0 | 48 | 50 | 1 | 1 | 482 | 26.2 | 3300 | 18.4 | 64.0 | 33.3 | 31.0 | 62.0 |
| C13 | 7.93 | 0 | | 8.7 | 4.8 | 12.7 | 39.8 | 83.0 | 26.7 | 32.0 | 24 | 67 | 4 | 5 | 507 | 187.2 | 5829 | 40.9 | 54.9 | 47.8 | 38.5 | 53.0 |
| C14 | 8.93 | 0 | | 5.0 | 4.6 | 11.4 | 36.5 | 79.0 | 24.7 | 31.2 | 47 | 51 | 2 | 0 | 299 | 194.4 | 2550 | 13.6 | 41.6 | 37.8 | 49.1 | 53.3 |
| R10 | 14 | 6.5 | 70 | 3.2 | 5.6 | 15.2 | 47.1 | 85.0 | 27.2 | 32.2 | 46 | 49 | 5 | 0 | 234 | 22.0 | 1568 | 24.5 | 52.8 | 15.4 | 35.0 | 35.2 |
| R11 | 14 | 6.5 | 66 | 8.3 | 5.9 | 14.0 | 44.9 | 77.0 | 23.9 | 31.2 | 43 | 54 | 3 | 0 | 351 | 57.0 | 4482 | 19.0 | 54.5 | 46.1 | 39.2 | 72.7 |
| R12 | 12 | 6.5 | 66 | 6.2 | 5.5 | 13.8 | 42.6 | 77.0 | 24.9 | 32.4 | 56 | 38 | 4 | 1 | 301 | 53.8 | 2356 | 13.7 | 63.2 | 31.8 | 32.5 | 51.4 |
| R13 | 13 | 6.5 | 70 | 5.8 | 5.3 | 13.0 | 39.7 | 74.0 | 24.4 | 32.8 | 33 | 60 | 4 | 2 | 429 | 46.6 | 3480 | 9.6 | 66.2 | 45.2 | 30.6 | 36.1 |
| R14 | 14 | 6.5 | 68 | 4.2 | 5.9 | 13.8 | 43.4 | 74.0 | 23.5 | 31.9 | 63 | 34 | 3 | 0 | 364 | 98.4 | 1428 | 9.3 | 58.7 | 28.1 | 37.3 | 16.3 |
| R15 | 15 | 6.5 | 68 | 6.9 | 6.2 | 14.3 | 45.4 | 73.0 | 23.1 | 31.5 | 53 | 45 | 1 | 1 | 354 | 326.0 | 3105 | 14.3 | 70.3 | 48.5 | 25.9 | 56.0 |
| R16 | 11 | 6.5 | 68 | 7.6 | 5.6 | 13.6 | 42.8 | 77.0 | 24.5 | 31.8 | 71 | 24 | 4 | 0 | 440 | 11.4 | 1824 | 16.6 | 49.3 | 32.1 | 45.8 | 51.2 |
| R17 | 13 | 6.5 | 66 | 7.4 | 5.2 | 13.4 | 42.2 | 81.0 | 25.7 | 31.7 | 34 | 62 | 4 | 0 | 440 | 142.6 | 4588 | 42.0 | 50.4 | 55.4 | 45.6 | 71.8 |
| R18 | 11 | 6.5 | 66 | 3.0 | 5.3 | 12.2 | 37.4 | 70.0 | 22.9 | 32.6 | 46 | 45 | 9 | 0 | 426 | 420.0 | 1350 | 9.2 | 65.1 | 12.1 | 25.0 | 33.0 |
| R19 | 6.36 | 6.75 | 48 | 8.2 | 5.6 | 12.3 | 39.2 | 70.0 | 22.1 | 31.5 | 39 | 52 | 6 | 4 | 286 | 1020.0 | 4264 | 22.5 | 56.6 | 61.0 | 38.8 | 75.8 |
| R20 | 6.36 | 6.75 | 49 | 5.1 | 4.7 | 12.0 | 39.1 | 83.0 | 25.5 | 30.8 | 41 | 45 | 4 | 11 | 376 | 252.0 | 2295 | 17.8 | 55.8 | 35.0 | 37.5 | 30.0 |
| R21 | 7 | 6.75 | 44 | 7.0 | 4.6 | 11.9 | 37.1 | 82.0 | 26.2 | 32.1 | 47 | 47 | 4 | 1 | 250 | 482.0 | 3290 | 24.2 | 53.2 | 66.3 | 41.3 | 81.7 |
| R22 | 7 | 6.75 | 46 | 6.5 | 4.7 | 11.4 | 36.4 | 78.0 | 24.5 | 31.4 | 45 | 50 | 3 | 2 | 374 | 258.0 | 3250 | 10.8 | 68.3 | 45.2 | 28.5 | 65.2 |
| R5 | 7.08 | 7.2 | 57 | 5.7 | 5.2 | 12.6 | 40.4 | 78.0 | 24.4 | 31.1 | 55 | 40 | 4 | 1 | 257 | 342.0 | 2280 | 1.9 | 46.8 | 35.1 | 49.4 | 50.8 |
| R23 | 6 | 7.2 | 45 | 9.3 | 4.8 | 12.4 | 39.8 | 82.0 | 25.6 | 31.1 | 71 | 23 | 4 | 2 | 319 | 92.2 | 2139 | 9.1 | 48.4 | 49.2 | 41.1 | 55.3 |
| R24 | 6 | 7.2 | 44 | 6.5 | 4.6 | 11.6 | 36.3 | 80.0 | 25.6 | 32.1 | 45 | 51 | 2 | 1 | 345 | 150.6 | 3315 | 13.7 | 55.3 | 43.0 | 35.2 | 64.1 |
| R25 | 6 | 7.2 | 44 | 4.7 | 5.3 | 12.7 | 40.4 | 76.0 | 23.9 | 31.4 | 31 | 65 | 3 | 1 | 339 | 942.0 | 3055 | 12.6 | 51.9 | 60.2 | 38.1 | 80.0 |
| R26 | 6 | 7.2 | 46 | 5.5 | 4.8 | 12.0 | 37.5 | 78.0 | 25.0 | 31.9 | 46 | 49 | 4 | 1 | 268 | 230.0 | 2695 | 4.6 | 66.5 | 46.0 | 28.4 | 60.4 |
| R1 | 8.08 | 7.55 | 57 | 9.3 | 5.1 | 12.9 | 40.9 | 80.0 | 25.4 | 31.6 | 61 | 30 | 3 | 6 | 306 | 254.0 | 2790 | 14.6 | 50.3 | 36.7 | 42.1 | 62.6 |
| R4 | 7.08 | 7.55 | 57 | 8.9 | 4.8 | 12.6 | 37.8 | 79.0 | 26.2 | 33.3 | 31 | 66 | 2 | 1 | 297 | 92.6 | 5874 | 23.1 | 55.4 | 74.6 | 41.8 | 79.6 |
| R27 | 6.36 | 7.55 | 49 | 13.7 | 4.9 | 11.9 | 37.3 | 76.0 | 24.5 | 32.1 | 80 | 17 | 1 | 2 | 236 | 314.0 | 2329 | 6.2 | 56.6 | 51.3 | 36.0 | 70.3 |
| R28 | 7 | 7.55 | 44 | 8.4 | 3.9 | 10.1 | 31.9 | 83.0 | 26.1 | 31.5 | 24 | 66 | 6 | 4 | 373 | 528.0 | 5544 | 16.5 | 51.7 | 56.6 | 40.6 | 53.9 |
| R29 | 6.9 | 7.55 | 44 | 8.1 | 4.9 | 11.7 | 38.2 | 78.0 | 23.6 | 30.5 | 40 | 54 | 4 | 3 | 414 | 226.0 | 4374 | 4.5 | 36.3 | 39.5 | 51.8 | 53.4 |
| R2 | 7.08 | 7.85 | 56 | 5.4 | 4.6 | 11.8 | 38.1 | 82.0 | 25.6 | 31.1 | 46 | 51 | 2 | 1 | 329 | 2340.0 | 2754 | 6.8 | 61.8 | 42.5 | 34.1 | 75.8 |
| R3 | 7.08 | 8.05 | 57 | 9.8 | 4.8 | 12.3 | 38.7 | 82.0 | 25.9 | 31.8 | 26 | 65 | 8 | 0 | 264 | 472.0 | 6370 | 14.1 | 45.9 | 57.1 | 49.5 | 63.2 |
| R30 | 6 | 8.05 | 46 | 11.1 | 4.9 | 11.4 | 36.7 | 75.0 | 23.4 | 31.1 | 22 | 74 | 4 | 0 | 278 | 516.0 | 8214 | 6.2 | 55.7 | 69.8 | 36.9 | 69.3 |
| R31 | 7 | 8.05 | 44 | 7.8 | 5.0 | 11.7 | 37.2 | 75.0 | 23.5 | 31.5 | 40 | 53 | 3 | 3 | 443 | 153.8 | 4134 | 3.1 | 49.0 | 36.2 | 44.5 | 75.4 |
| R32 | 6 | 8.05 | 46 | 7.2 | 5.0 | 12.6 | 39.9 | 81.0 | 25.5 | 31.6 | 62 | 32 | 3 | 4 | 256 | 162.0 | 2304 | 3.0 | 61.9 | 57.7 | 32.1 | 56.0 |
| R9 | 7.83 | 8.4 | 54 | 10.4 | 5.3 | 12.8 | 40.5 | 76.0 | 23.9 | 31.6 | 42 | 50 | 3 | 4 | 243 | 125.2 | 5200 | 7.7 | 44.4 | 52.8 | 34.1 | 63.1 |
