# Supplementary material for: Late effects of total body irradiation on hematopoietic recovery and immune function in rhesus macaques
Source: PLoS One. 2019 Feb 13;14(2):e0210663. doi: 10.1371/journal.pone.0210663 (PMC6373904; doi:10.1371/journal.pone.0210663)
Supplement: S2 Table — (PDF) [file pone.0210663.s002.pdf]

Vaccine responses against tetanus and rabies (raw data)

Tetanus IgG (units/ml)	R1	R2	R3	R4	R5	R6	R7	R8	R9	C3	C4	C1	C2
week 1	2512	2935	2991	3867	1988	2727	3054	2078	2400	2647	2406	3447	2959
week 2	2697		2991	3552	2263	2632	3247	2273	2316	4451	2074	3785	2820
week 3	2683	2825	3964	3544	2424	2528	3394	2108	2422	5587	2424	4123	2999
week 4	2563	3428	4477	3562	2376	4109	3162	2271	2786	8172	2852	4078	2717
week 5	4232	3890	6716	4843	2656	4695	3298	2830	3102	9637	2558	4245	3912
week 6	3943	4205	7811	4430	2839	5019	3551	3835	3146	8559	2376	4061	4790
week 7	4534	4510	7109	4912	2479	5397	3305	3465	3128	9050	2487	4569	5894
week 8	5109	5471	7432	4108	2871	5562	3720	3655	3189	6786	1049	4689	6496
week 9	6487	4581	7532	4708	3457	6530	4602	3328	2999	9254	2263	4347	6514
week 10	7866	4336	7796	4811	3144	8244	3708	3676	3255	7969	3129	4624	6472
week 11	6502	4450	10506	5060	3012	9124	4136	3265	3197	8450	2832	4938	6585
week 12	5358	2876	5686	3402	1517		2389	1814	2120	8302	2569	2971	5864
week 13	7691	4217	8086	5431	3358		3928	2675	3102	8113	2604	4696	6321
week 14	7224	4745	8461	5366	2935		4047	3064	3678	6855	2561	4734	6365
week 15	7604	4286	7637	4937	3242		4341	2914	3443	9148	2571	5037	5850
week 16	8479	4721	6848	4960	2882		4237	2525	4066	7851	2382	5038	5834

Tetanus IgM (units/ml)	R1	R2	R3	R4	R5	R6	R7	R8	R9	C3	C4	C1	C2
week 1	104158	21124	50202	67432	113410	85633	20883	43324	39026	117980	45033	106740	89183
week 2	73120	57356	55440	59727	91128	67385	72452	61620	38131	138260	80625	179460	99033
week 3	70658	59979	69858	59369	117000	73593	103580	53121	48032	112500	47462	178710	116240
week 4	80014	57660	80272	81532	113790	79161	56413	94205	48573	118810	66774	200000	122720
week 5	66888	71255	64304	116880	125220	73182	61391	67064	45546	121240	51610	174200	112190
week 6	103420	99249	90022	71704	113500	70689	50684	89526	43970	120230	53873	193220	92667
week 7	94596	83828	68644	63555	111190	84734	61849	51018	41669	109400	54323	219140	95154
week 8	85984	96203	62130	81907	90069	76675	58169	67196	41669	112660	60099	169320	107620
week 9	74570	59682	50678	62540	85837	69481	56468	41248	56065	116490	41576	145820	93184
week 10	85564	64004	64386	72384	101260	77113	48379	66126	47143	106680	36752	155440	98999
week 11	87190	78298	62636	70866	95007	85474	53753	50372	48337	117120	50883	161920	109110
week 12	73912	64217	58188	91559	91217		53725	62162	60704	127560	40113	182910	110830
week 13	90094	66661	85512	107830	104840		64592	44748	62127	145800	50772	157050	93647
week 14	79226	74761	87786	91141	98749		68654	59377	59572	107560	56343	138160	91965
week 15	79894	66778	67418	85825	99288		59870	59523	59620	159520	67481	150150	102100
week 16	85390	62814	53218	75116	85738		51406	148920	52920	112630	67188	140560	80688

Rabies IgG + IgM (EU/ml)	R1	R2	R3	R4	R5	R6	R7	R8	R9	C3	C4	C1	C2
week 1	0	0	0	0	0	0	0	0	0	0	163	0	0
week 2	0	0	0	0	0	0	0	0	0	0	111	0	0
week 3	0	0	0	0	0	0	0	0	0	0	120	0	0
week 4	469	302	677	215	174	317	334	180	101	0	97	195	351
week 5	690	480	1156	276	255	454	397	316	196	2456	99	253	299
week 6	1100	610	1259	392	387	573	1100	506	228	1815	89	274	281
week 7	1100	693	1736	393	1100	608	390	1270	256	1100	85	282	281
week 8	1100	605	2407	387	1100	588	255	951	229	1100	153	330	350
week 9	1100	687	1827	355	311	485	212	647	199	1100	79	227	251
week 10	1100	604	1193	277	300	591	194	528	188	1100	89	183	198
week 11	1058	711	1522	244	257	482	221	348	245	1226	104	156	182
week 12	891	607	1083	220	249	520	210	319	212	863	106	187	199
week 13	715	485	1055	202	334	588	209	999	196	955	130	176	212
week 14	1100	480	1391	182	384	564	168	369	152	1516	177	184	177
week 15	825	525	1119	182	1100	489	1100	203	104	901	224	207	182
week 16	633	590	1451	175	363	554	172	545	139	1671	252	195	184
