# Supplementary figures and images for: Late effects of total body irradiation on hematopoietic recovery and immune function in rhesus macaques
Source: PLoS One. 2019 Feb 13;14(2):e0210663. doi: 10.1371/journal.pone.0210663 (PMC6373904; doi:10.1371/journal.pone.0210663)

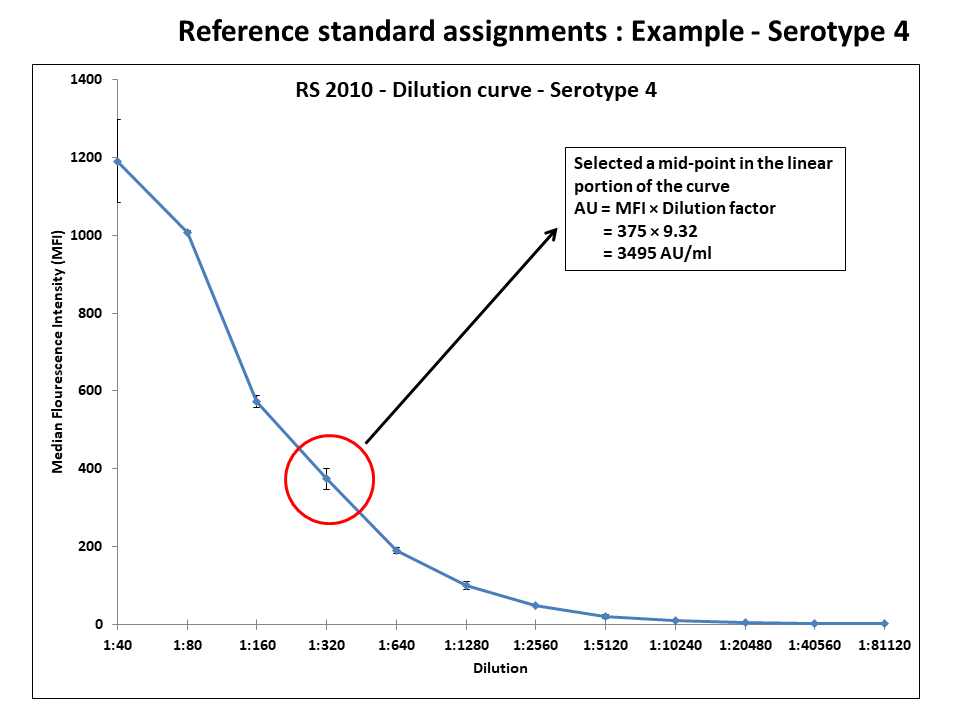

Supplement: S1 Fig — Reactivity of RS2010 with each pneumococcal serotype polysaccharide was used to define IgG responses to that serotype in arbitrary units (AU), defined as median fluorescence intensity in the Luminex-based immunoassay x dilution factor, as shown here for serotype 4. (TIF) [file pone.0210663.s003.tif]
