# Supplementary material for: Late effects of total body irradiation on hematopoietic recovery and immune function in rhesus macaques
Source: PLoS One. 2019 Feb 13;14(2):e0210663. doi: 10.1371/journal.pone.0210663 (PMC6373904; doi:10.1371/journal.pone.0210663)
Supplement: S2 Fig — The mean IgG level and standard error of the mean (n = 3 replicates) is plotted in arbitrary units for each animal relative to reference serum RS2010 for each of 11 pneumococcal serotypes, as described in the Materials and Methods. To be considered a positive reaction to vaccination, the IgG level measured at week 16 post-vaccination had to be significantly increased relative to the IgG level measured at week 0, prior to vaccination. Designation of each response as positive (+) or negative (-) is indicated for each animal and serotype combination. A. Serotype 1. B. Serotype 3. C. Serotype 4. D. Serotype 5. E. Serotype 6B. F. Serotype 7F. G. Serotype 9V. H. Serotype 14. I. Serotype 18C. J. Serotype 19F. K. Serotype 23F. These results were summarized in Table 1 of the manuscript. (PDF) [file pone.0210663.s004.pdf]

A.

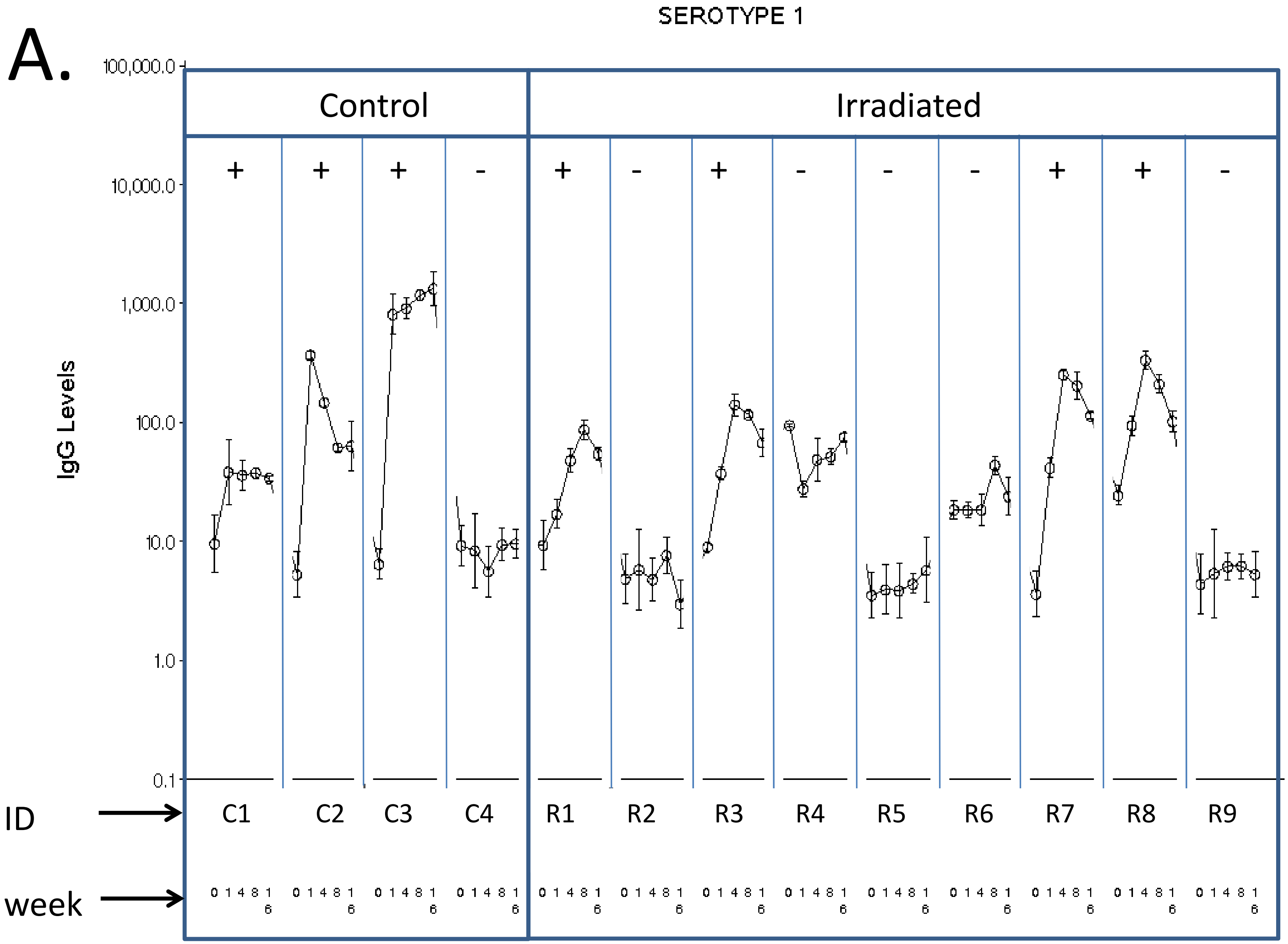
SEROTYPE 1
IgG Levels
100,000.0
10,000.0
1,000.0
100.0
10.0
1.0
0.1
Control
Irradiated
+ + + - + - + - - - + + -
ID
C1 C2 C3 C4 R1 R2 R3 R4 R5 R6 R7 R8 R9
week
0 1 4 8 16

B.

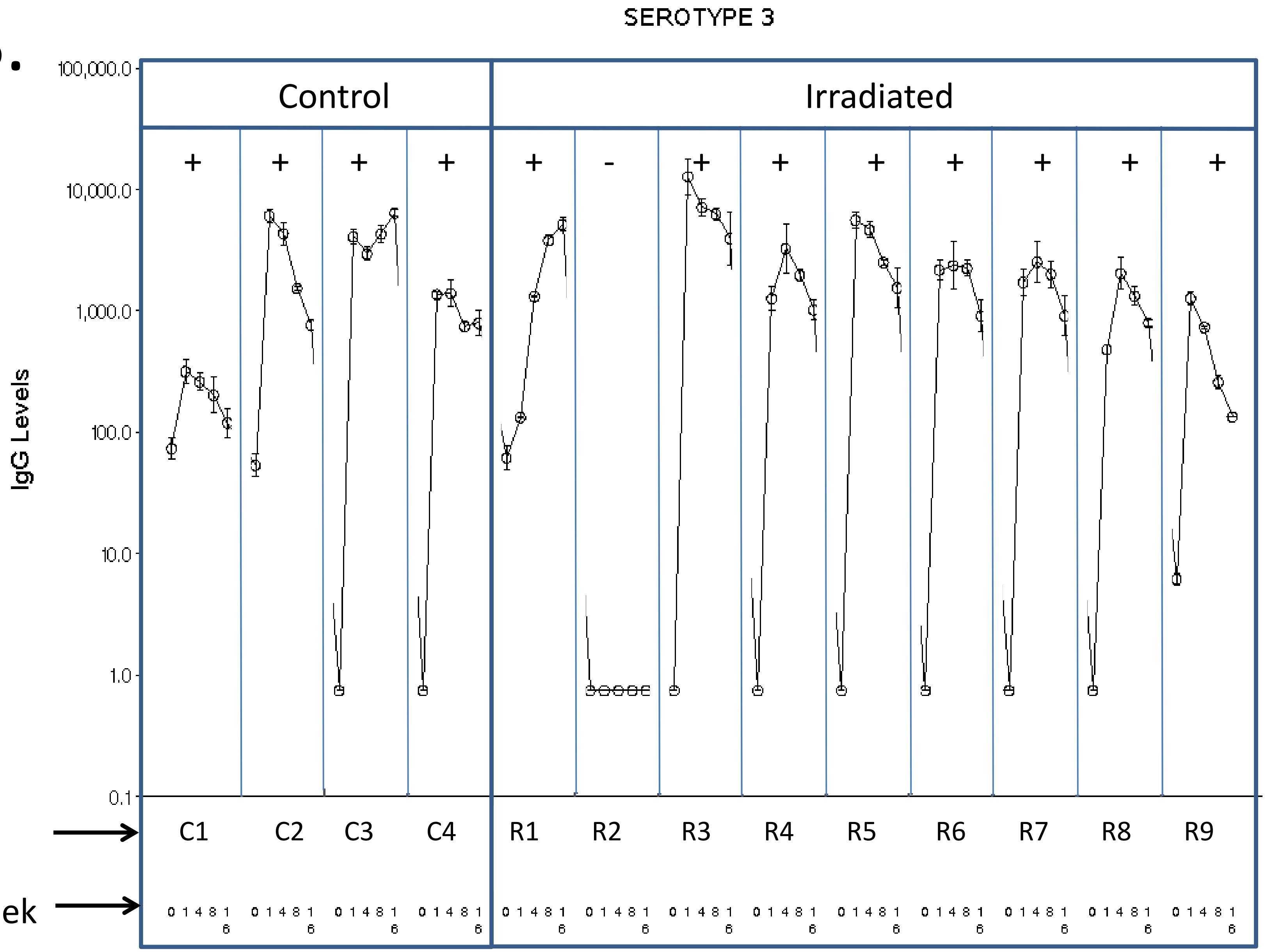
SEROTYPE 3
100,000.0
10,000.0
1,000.0
100.0
10.0
1.0
0.1
IgG Levels
Control
Irradiated
+ + + + + - + + + + + + +
ID
week
C1 C2 C3 C4 R1 R2 R3 R4 R5 R6 R7 R8 R9
0 1 4 8 16

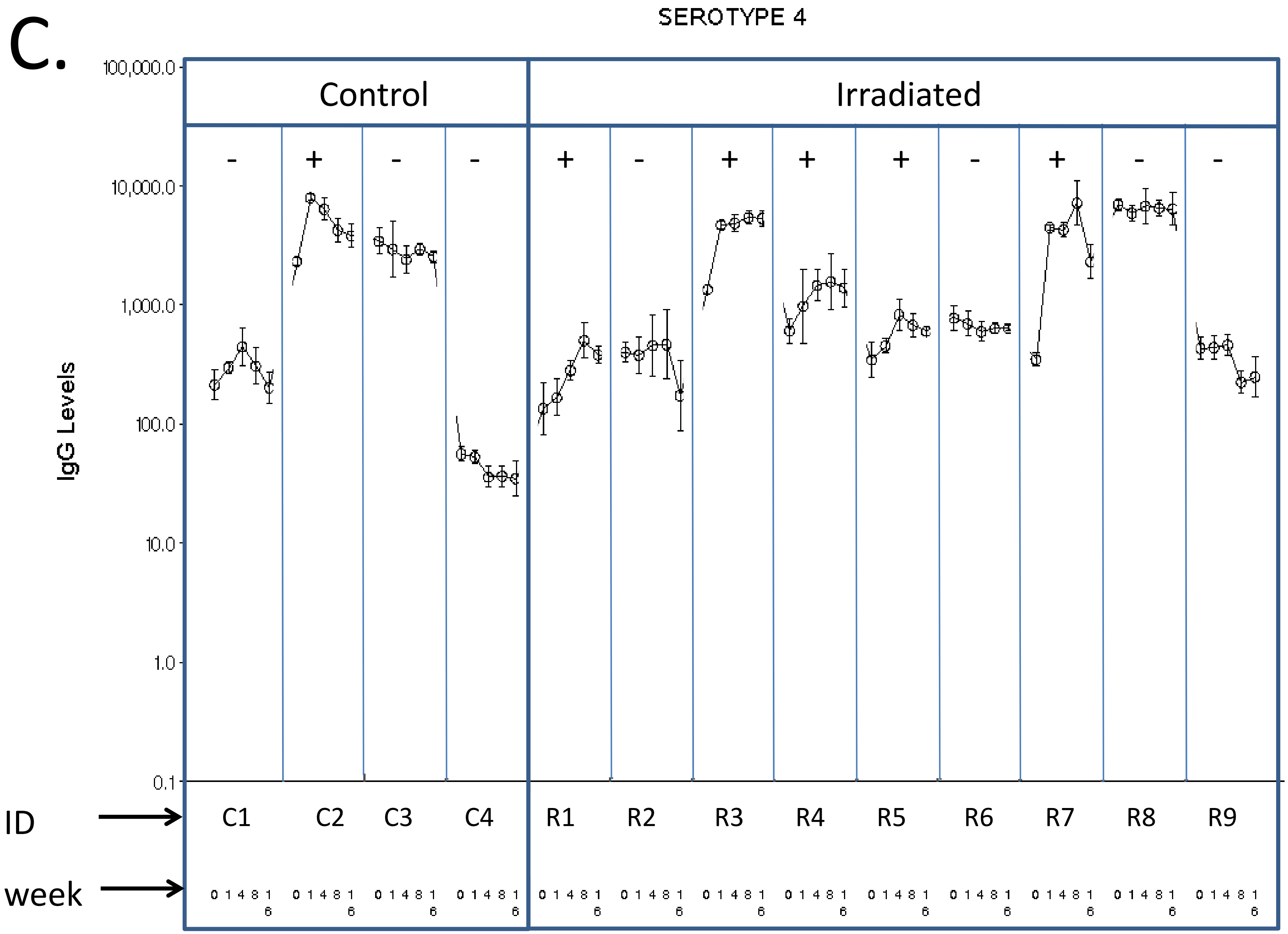
C.
SEROTYPE 4
Control
Irradiated
IgG Levels
100,000.0
10,000.0
1,000.0
100.0
10.0
1.0
0.1
- + - - + - + + + - + - -
ID
C1 C2 C3 C4 R1 R2 R3 R4 R5 R6 R7 R8 R9
week
0 1 4 8 16

D.

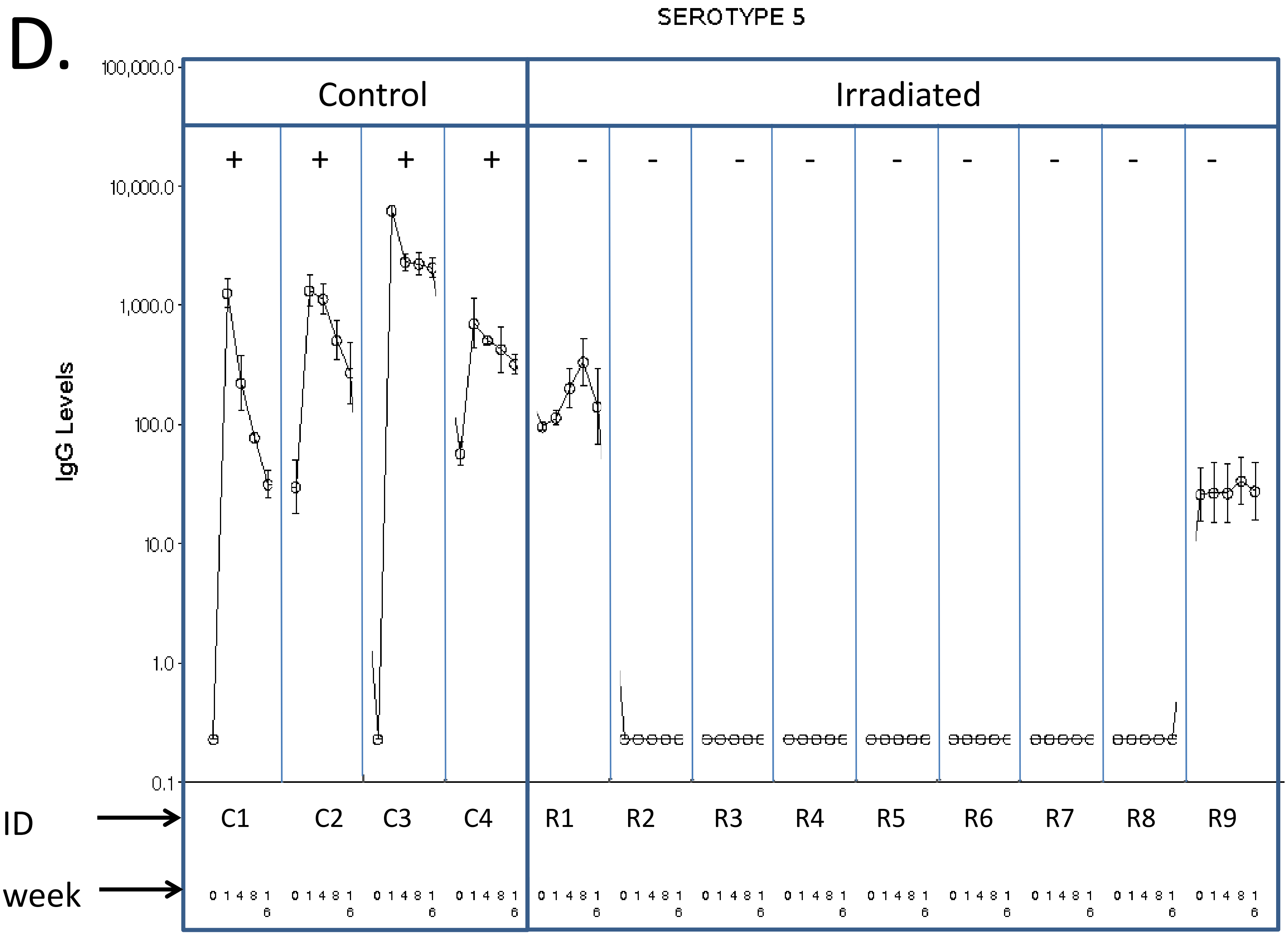

SEROTYPE 5
Control
Irradiated
IgG Levels
100,000.0
10,000.0
1,000.0
100.0
10.0
1.0
0.1
+
+
+
+
-
-
-
-
-
-
-
-
-
ID
C1
C2
C3
C4
R1
R2
R3
R4
R5
R6
R7
R8
R9
week
0 1 4 8 16

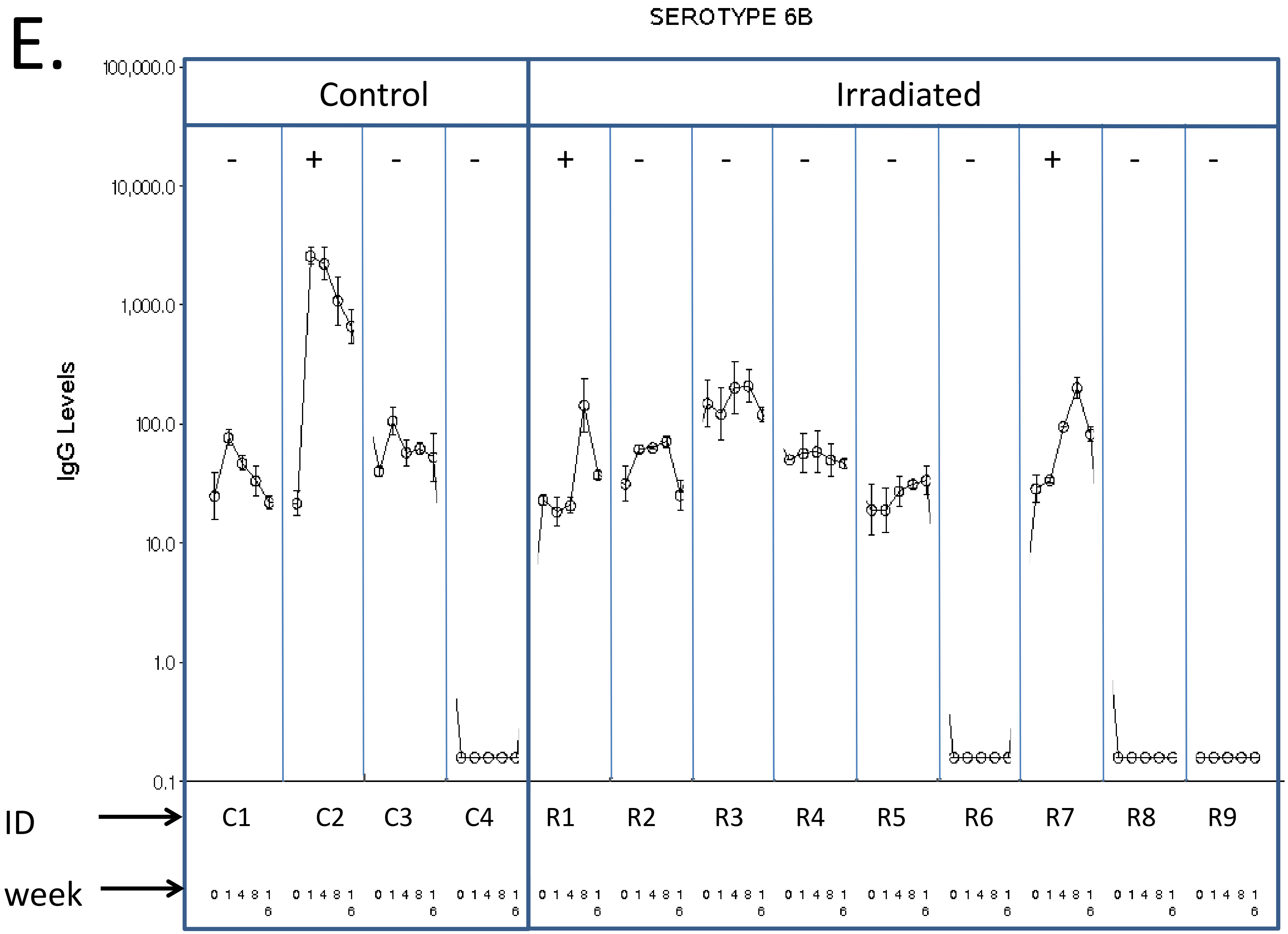
E.
SEROTYPE 6B
Control
Irradiated
IgG Levels
100,000.0
10,000.0
1,000.0
100.0
10.0
1.0
0.1
- + - - + - - - - - + - -
ID
C1 C2 C3 C4 R1 R2 R3 R4 R5 R6 R7 R8 R9
week
0 1 4 8 16

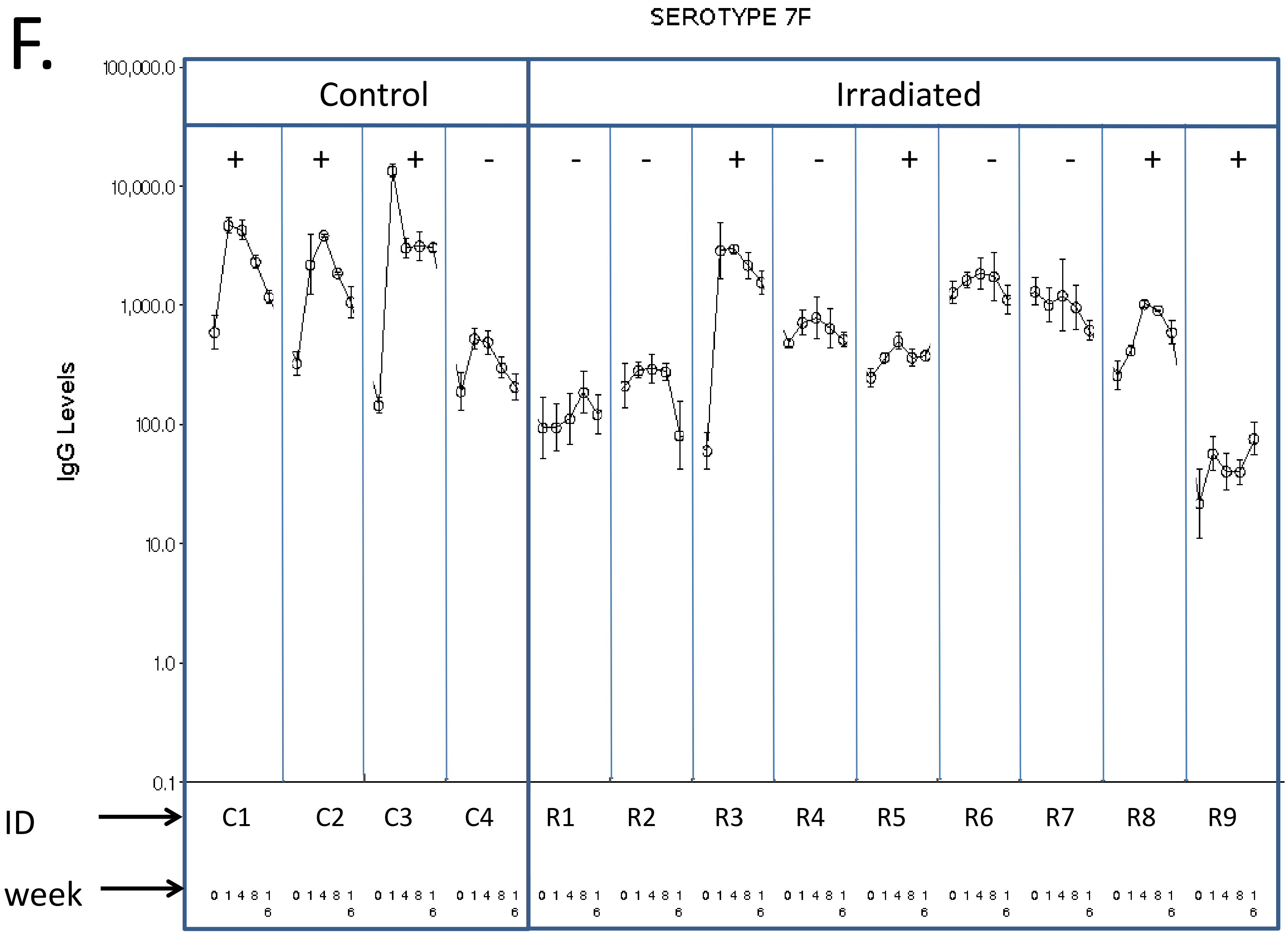
F.
SEROTYPE 7F
Control
Irradiated
IgG Levels
100,000.0
10,000.0
1,000.0
100.0
10.0
1.0
0.1
+ + + - - - + - + - - + +
ID
C1 C2 C3 C4 R1 R2 R3 R4 R5 R6 R7 R8 R9
week
0 1 4 8 16

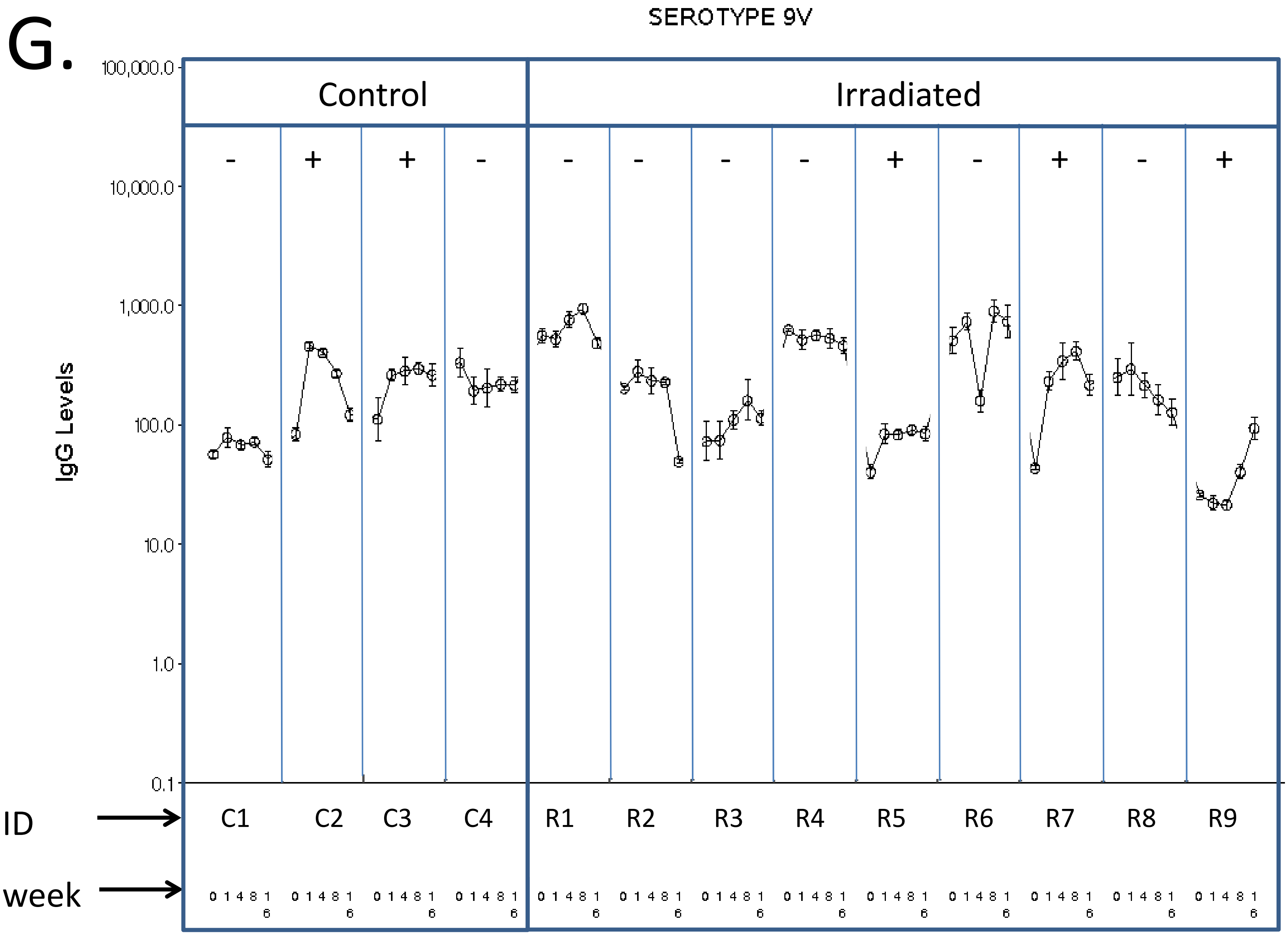
G.
SEROTYPE 9V
IgG Levels
100,000.0
10,000.0
1,000.0
100.0
10.0
1.0
0.1
Control
Irradiated
- + + - - - - - + - + - +
ID
C1 C2 C3 C4 R1 R2 R3 R4 R5 R6 R7 R8 R9
week
0 1 4 8 16

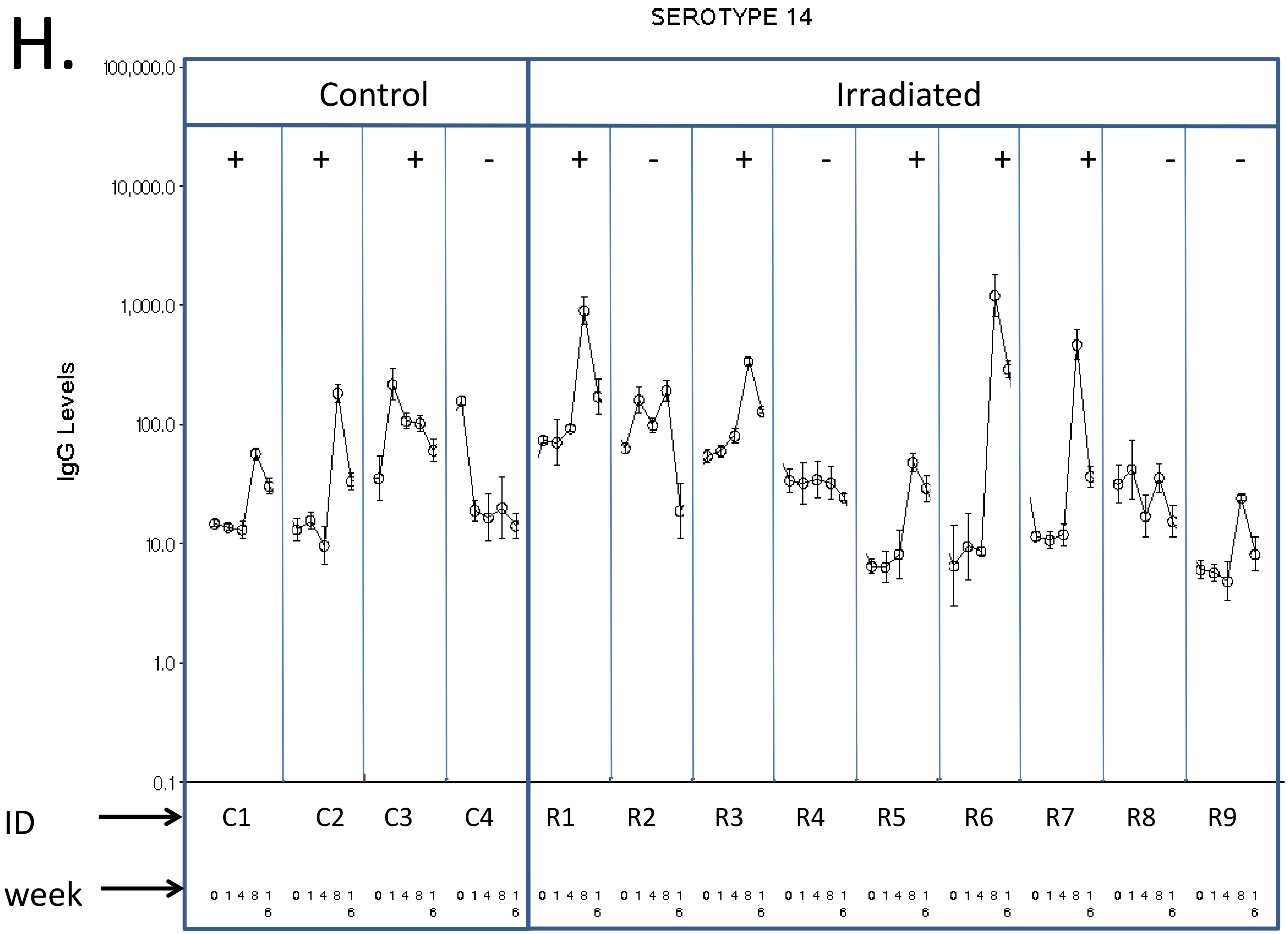

H.
SEROTYPE 14
Control
Irradiated
IgG Levels
100,000.0
10,000.0
1,000.0
100.0
10.0
1.0
0.1
+ + + - + - + - + + + - -
ID
C1 C2 C3 C4 R1 R2 R3 R4 R5 R6 R7 R8 R9
week
0 1 4 8 16

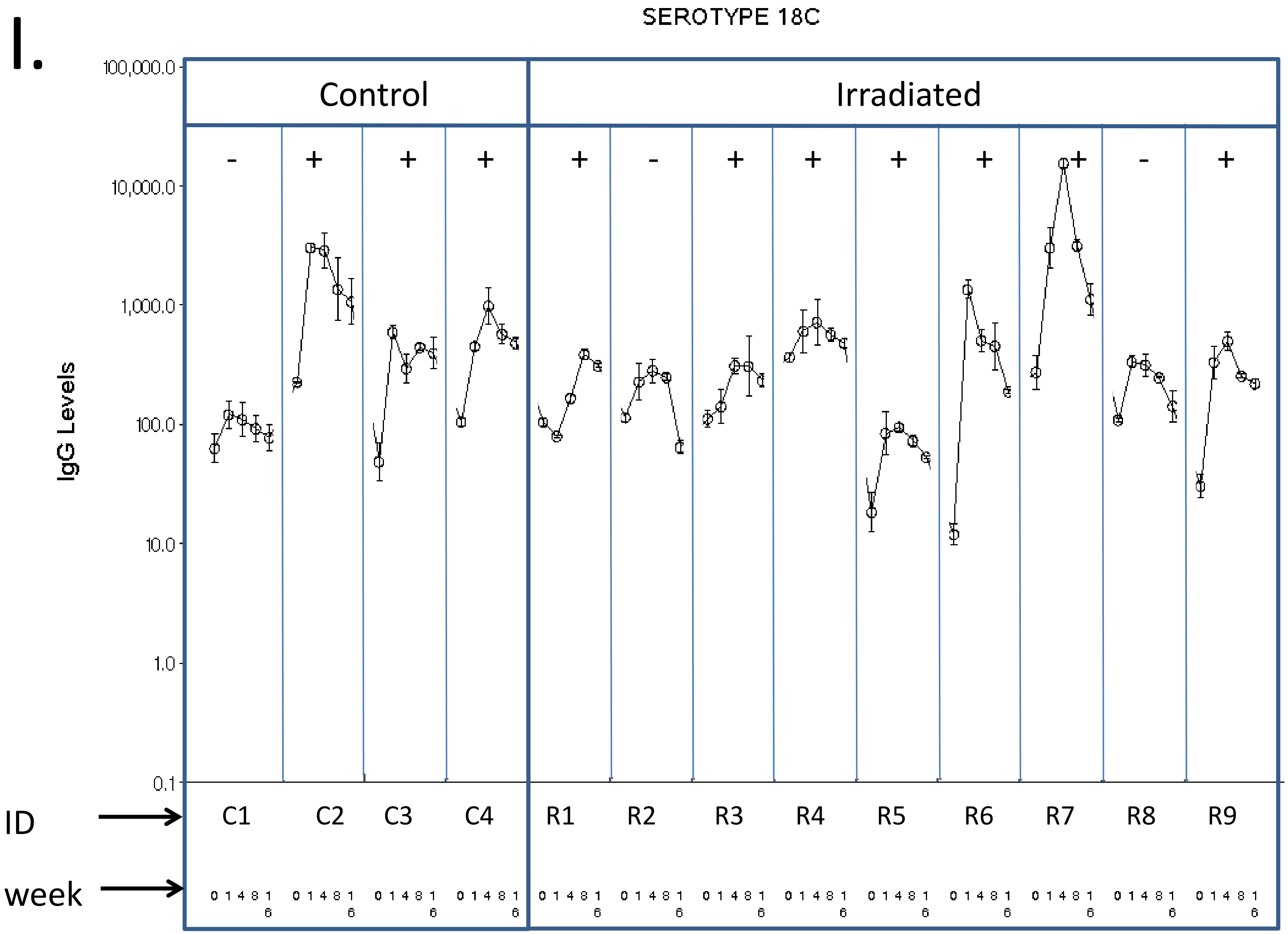

I.
SEROTYPE 18C
IgG Levels
100,000.0
10,000.0
1,000.0
100.0
10.0
1.0
0.1
Control
Irradiated
- + + + + - + + + + + - +
ID
week
C1 C2 C3 C4 R1 R2 R3 R4 R5 R6 R7 R8 R9
0 1 4 8 1 6

J.

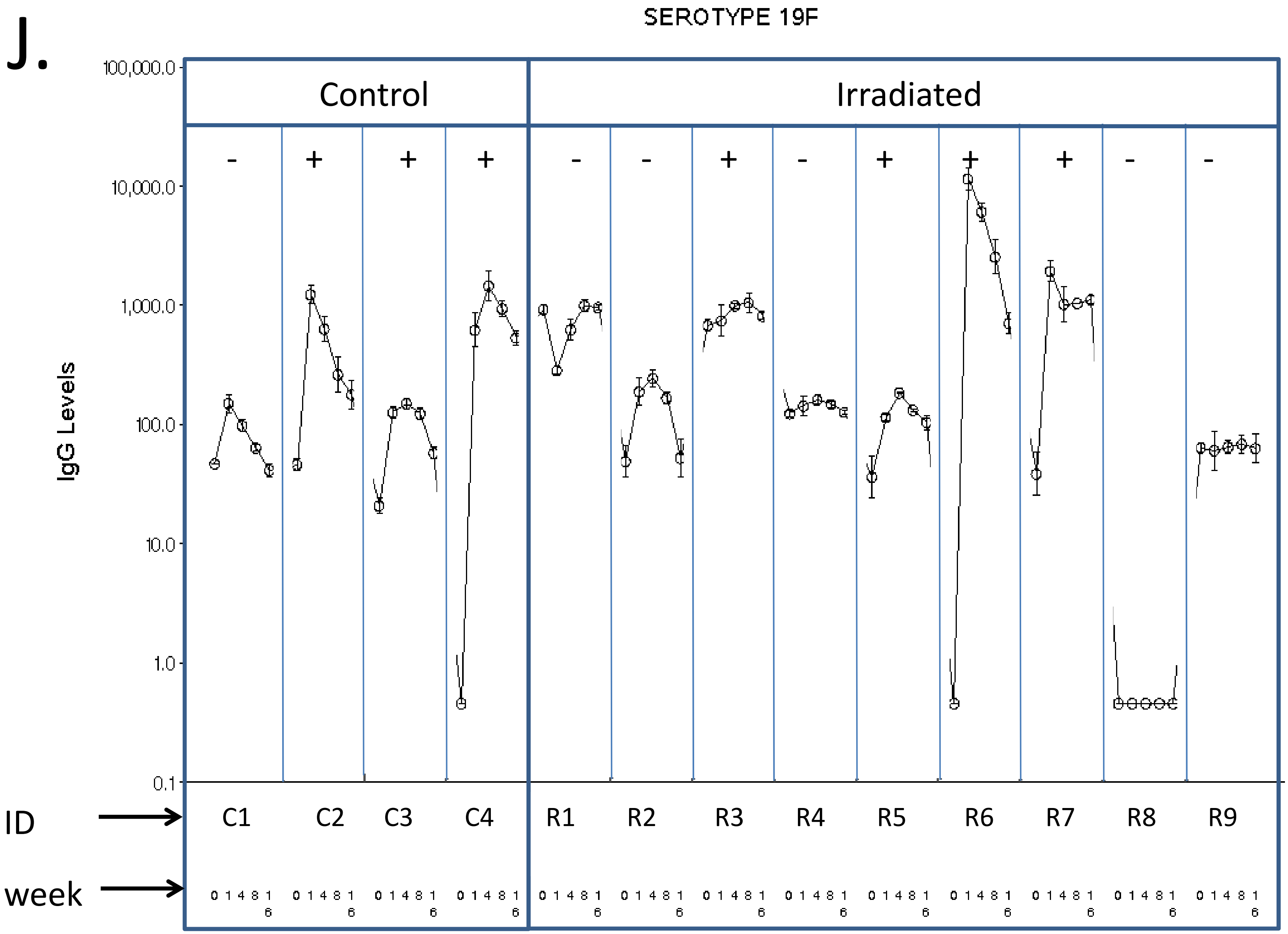
SEROTYPE 19F
Control
Irradiated
IgG Levels
100,000.0
10,000.0
1,000.0
100.0
10.0
1.0
0.1
- + + + - - + - + + + - -
ID
C1 C2 C3 C4 R1 R2 R3 R4 R5 R6 R7 R8 R9
week
0 1 4 8 16

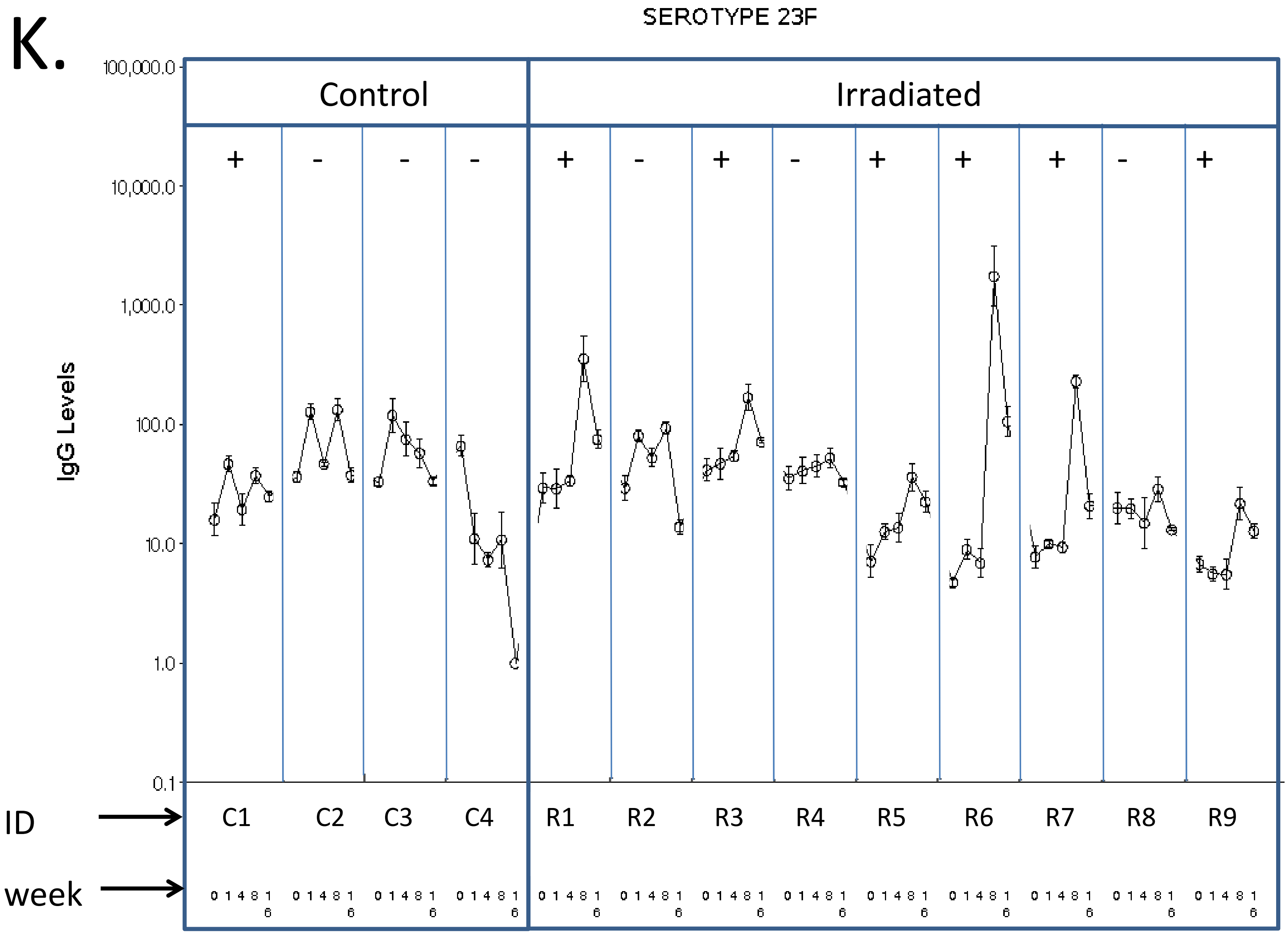

K.
SEROTYPE 23F
Control
Irradiated
IgG Levels
100,000.0
10,000.0
1,000.0
100.0
10.0
1.0
0.1
+
-
-
-
+
-
+
-
+
+
+
-
+
ID
week
C1
C2
C3
C4
R1
R2
R3
R4
R5
R6
R7
R8
R9
0 1 4 8 16
